# Clinical Implications and Molecular Features of Extracellular Matrix Networks in Soft Tissue Sarcomas

**DOI:** 10.1158/1078-0432.CCR-23-3960

**Published:** 2024-05-29

**Authors:** Valeriya Pankova, Lukas Krasny, William Kerrison, Yuen B. Tam, Madhumeeta Chadha, Jessica Burns, Christopher P. Wilding, Liang Chen, Avirup Chowdhury, Emma Perkins, Alexander T.J. Lee, Louise Howell, Nafia Guljar, Karen Sisley, Cyril Fisher, Priya Chudasama, Khin Thway, Robin L. Jones, Paul H. Huang

**Affiliations:** 1 Division of Molecular Pathology, The Institute of Cancer Research, London, United Kingdom.; 2 Precision Sarcoma Research Group, German Cancer Research Center (DKFZ), Heidelberg, Germany.; 3 National Center for Tumor Diseases, Heidelberg, Germany.; 4 The Christie NHS Foundation Trust, Manchester, United Kingdom.; 5 Light Microscopy Facility, The Institute of Cancer Research, London, United Kingdom.; 6 Division of Clinical Medicine, The Medical School, University of Sheffield, Sheffield, United Kingdom.; 7 University Hospitals Birmingham NHS Foundation Trust, Birmingham, United Kingdom.; 8 The Royal Marsden NHS Foundation Trust, London, United Kingdom.; 9 Division of Clinical Studies, The Institute of Cancer Research, London, United Kingdom.

## Abstract

**Purpose:**

The landscape of extracellular matrix (ECM) alterations in soft tissue sarcomas (STS) remains poorly characterized. We aimed to investigate the tumor ECM and adhesion signaling networks present in STS and their clinical implications.

**Experimental Design:**

Proteomic and clinical data from 321 patients across 11 histological subtypes were analyzed to define ECM and integrin adhesion networks. Subgroup analysis was performed in leiomyosarcomas (LMS), dedifferentiated liposarcomas (DDLPS), and undifferentiated pleomorphic sarcomas (UPS).

**Results:**

This analysis defined subtype-specific ECM profiles including enrichment of basement membrane proteins in LMS and ECM proteases in UPS. Across the cohort, we identified three distinct coregulated ECM networks which are associated with tumor malignancy grade and histological subtype. Comparative analysis of LMS cell line and patient proteomic data identified the lymphocyte cytosolic protein 1 cytoskeletal protein as a prognostic factor in LMS. Characterization of ECM network events in DDLPS revealed three subtypes with distinct oncogenic signaling pathways and survival outcomes. Evaluation of the DDLPS subtype with the poorest prognosis nominates ECM remodeling proteins as candidate antistromal therapeutic targets. Finally, we define a proteoglycan signature that is an independent prognostic factor for overall survival in DDLPS and UPS.

**Conclusions:**

STS comprise heterogeneous ECM signaling networks and matrix-specific features that have utility for risk stratification and therapy selection, which could in future guide precision medicine in these rare cancers.

**Translational Relevance**

The extracellular matrix (ECM) has been shown in some epithelial cancers to be a rich source of drug targets and prognostic biomarkers but is poorly characterized in mesenchymal tumors such as sarcomas. In this study, we analyze the ECM networks in a cohort of 321 soft tissue sarcomas (STS) and their association with clinicopathological variables and survival outcomes. We find that STS are defined by histotype-specific ECM profiles and coregulated networks. ECM features such remodeling proteins are candidate antistromal drug targets for patients with dedifferentiated liposarcomas (DDLPS) with the poorest survival outcomes. Furthermore, we show that a proteoglycan signature is associated with overall survival in multivariable analysis in patients with DDLPS and undifferentiated pleomorphic sarcoma. This study demonstrates that information derived from ECM networks may help inform prognostication and select novel therapies in multiple STS subtypes.

## Introduction

Despite efforts to improve patient outcomes in soft tissue sarcomas (STS), the median 5-year survival rate has remained at 60%, reducing to 10% in patients with advanced disease (1). This challenge is further compounded by the inherent molecular heterogeneity associated with the >150 histological subtypes with distinct underlying genetics. Many of the prior studies investigating the biology of sarcomas, including large-scale genomic, transcriptomic, and epigenetic analyses, have focused on the tumor cell component and do not fully consider the influence of the tumor microenvironment (TME) and specifically the extracellular matrix (ECM). While such studies have been impactful in furthering our understanding of a subset of molecular drivers in STS, they only represent one facet of the evolving tumor landscape in disease progression and therapy failure. In particular, the composition of the ECM in the TME and its effects on tumor cell biology in STS is largely unknown and remains a fundamental gap in our knowledge of these rare mesenchymal tumors (2).

It is well established that the ECM plays important functional roles in driving multiple cancer hallmarks (3), primarily through bidirectional crosstalk between the surrounding ECM and their cognate integrin adhesion receptors on tumor and immune cells (4, 5). Dysregulation in matrix remodeling and integrin signaling has been implicated in cancer development and conceptually, targeted disruption of these ECM processes has the potential to deliver new therapies. Notably, the ECM has been shown in some epithelial cancers to be a rich source of drug targets and prognostic biomarkers. For instance, a number of transcriptomic-based matrix scores have been developed for risk stratification in lung and ovarian cancers (6, 7). A deep understanding of the ECM in STS therefore holds the promise of delivering a range of personalized oncology strategies.

Here we undertake a comprehensive analysis of the ECM composition across multiple histologies and examine the principles underpinning coordinated regulation of the matrix and integrin adhesion networks in this group of diseases. Functional studies using patient-derived ECM further illustrate the importance of the stroma in modulating STS cellular phenotypes. By harnessing ECM remodeling as a tool to dissect the biological heterogeneity of STS, we provide new avenues for molecular subtyping and risk stratification, which could be important in the future clinical management of sarcoma patients.

## Materials and Methods

### Patient cohort

This study comprises a cohort of 321 patients with STS from the Royal Marsden Hospital, National Taiwan University Hospital, and Newcastle University that has previously been reported by our laboratory (8). Retrospective collection and analysis of associated clinical data was approved as part of the Royal Marsden Hospital PROgnoStic and PrEdiCTive ImmUnoprofiling of Sarcomas (PROSPECTUS) study (CCR 4371, REC 16/EE/0213). Baseline clinicopathological characteristics and survival data were collected by retrospective review of medical records (8). Use of archival formalin-fixed, paraffin-embedded (FFPE) tumor samples and associated clinical data for immunohistochemical analyses was approved by Royal Marsden Hospital Institutional Review Board as part of the PROSPECTUS study and the study was conducted in accordance with the Declaration of Helsinki. Written informed consent was obtained from patients.

### Proteomic data of specimens from patients with STS

Proteomic data of 321 patient specimens were downloaded from ProteomeXchange (PXD036226; https://www.ebi.ac.uk/pride/archive/projects/PXD036226; ref. 8). Details of proteomic data processing are provided in the Supplementary Methods S1. The dataset was filtered for matrisome and adhesome proteins as defined according to MatrisomeDB (9) and the functional atlas of the adhesome (4, 5), respectively. Heatmaps of matrisome and adhesome expression matrices were generated using the ComplexHeatmap package (RRID:SCR_017270) in R for visualization. Details of processing and analysis of patient proteomic data are provided in the Supplementary Methods S1.

### Generation of leiomyosarcoma ECM solution

Patient samples were selected for inclusion based on availability of flash frozen tissue from primary excision extremity samples with histopathologically confirmed leiomyosarcoma (LMS) diagnosis. Seven LMS tumor specimens were obtained from the Royal Marsden Hospital NIHR BRC Biobank as part of the PROSPECTUS study. The decellularization protocol was adapted from Xu and colleagues (10). Briefly, tumor specimens were subjected to three freeze–thaw cycles. The tumors were cut into 2–3 mm^3^ pieces and washed in phosphate-buffered saline (PBS) solution at 25°C for 24 hours. Tumors were then incubated in a detergent solution [0.1% w/v of sodium dodecyl sulfate (SDS); Sigma-Aldrich], 10 mmol/L tris(hydroxymethyl)aminomethane (Tris) buffer pH 8.0, 0.1% v/v ethylenediaminetetraacetic acid (EDTA; Sigma-Aldrich) and 1× Halt Protease and Phosphatase Inhibitor Cocktail (Thermo Fisher Scientific) at 4°C for 4 days until the tumor pieces appeared white. The detergent solutions were agitated and changed every 8 to 16 hours. Tumors were incubated in PBS at 4°C for 24 hours to remove the detergent and cell debris. Tissue was then submerged in DNase solution (30 μg/mL; BD) in 1.3 mmol/L MgSO_4_ and 2 mmol/L CaCl_2_ at 25°C for 1 hour and then thoroughly rinsed with PBS for 24 hours. The resulting decellularized scaffolds were dried in a SpeedVac concentrator (Thermo Scientific).

The protocol for generating LMS ECM solution was adapted from Nehrenheim and colleagues (11). A total of 10 mg of decellularized scaffolds were digested with 1 mg/mL pepsin (Sigma-Aldrich) in 0.01 mol/L hydrochloric acid. The mixture was then mechanically homogenized in 0.5 mL tubes with ceramic beads (1.4 mm diameter, Bertin) at 6,800 rpm (three cycles of 20 seconds, with a 30-second break between cycles) using the Precellys Evolution homogenizer system (Bertin). The homogenized solution was left at 25°C for 48 hours to digest. The solubilized ECM solution was chilled on ice and neutralized with cold 10% v/v 0.1 mol/L NaOH and 11.1% v/v 10× PBS to pH 7. Details for proteomic analysis of LMS tumors and ECM solution are provided in the Supplementary Methods S1.

### Cell lines

SK-UT-1 and SK-UT-1b cells were obtained from the ATCC (RRID:CVCL_0533, CVCL_2250) and cultured in Minimum Essential Medium (MEM; Gibco) supplemented with 0.5% penicillin/streptomycin and 10% fetal bovine serum (FBS). Sheffield LMS-01 W1 (SHEF-LMS w1) and Sheffield LMS-01 WS (SHEF-LMS ws) cells (12) were obtained from Dr. Karen Sisley via the University of Sheffield sarcoma cell line repository and cultured in Roswell Park Memorial Institute (RPMI) 1640 supplemented with 0.5% penicillin/streptomycin, 10% FBS, 1% v/v of 240 mmol/L L-glutamine, and 0.4% v/v of 1 mol/L D-glucose. The ICR-LMS-1 cell line was established in our laboratory and grown in Dulbecco’s Modified Eagle Medium/Nutrient Mixture F-12 (DMEM:Ham’s F12 1:1) + 15 mmol/L N-2-hydroxyethylpiperazine-N′-2-ethanesulfonic acid (HEPES), supplemented with 0.5% penicillin/streptomycin, 10% FBS, 1% v/v of 240 mmol/L L-glutamine, 5 μg/mL of bovine insulin (Sigma), 0.4 μg/mL hydrocortisone (Sigma), 10 ng/mL epidermal growth factor (Peprotech), 250 ng/mL amphotericin B (Thermo Fisher Scientific), 9.6 ng/mL cholera toxin (Sigma), and 5 µmol/L Y-27632 dihydrochloride (LC labs). Details for the generation of the ICR-LMS-1 cell line and proteomic analysis of cell lines are provided in the Supplementary Methods S1. Before experiments, cell lines were authenticated by short tandem repeat DNA profiling (Eurofins Genomics) and tested for mycoplasma with a MycoStrip kit (InvivoGen). All cells were cultured in 5% CO_2_ at 37°C in humidified incubators.

### Live-cell imaging

Human histone H2B fused to green fluorescent protein (H2B-GFP) transduced SK-UT-1, ICR-LMS-1, SHEF-LMS w1, SHEF-LMS ws, and SK-UT-1b were seeded into plastic microplates coated with 0.1 mol/L acetic acid or plastic precoated with 10 μg/mL collagen IV (C5533, Sigma LOT109K3801), patient-derived LMS ECM solution diluted in ice-cold 0.1 mol/L acetic acid or fibronectin (F1141, Sigma LOT SLCD2908), laminin (CC095, Merck, LOT 3660319) diluted in ice-cold PBS, according to the manufacturer’s instruction. After 2 hours of incubation at 37°C and 5% CO_2_, the coating solution was aspirated, the plates were washed with PBS, and the cells were seeded. Details for lentiviral transduction of H2B-GFP are provided in the Supplementary Methods S1. Dried decellularized scaffolds from seven LMS patient samples were pooled in equal mass ratio and digested with pepsin to generate a pooled LMS ECM solution. Prior to undertaking a migration assay, PhenoPlate 96-well plastic microplates (Perkin Elmer) were coated with 10 μg/mL LMS ECM solution diluted in ice-cold 0.1 mol/L acetic acid. The plastic control condition was coated with 0.1 mol/L acetic acid. After 2 hours of incubation at 37°C and 5% CO_2_, the coating solution was aspirated, the plates were washed with PBS, and the cells were seeded.

Live-cell imaging was performed with a confocal laser scanning microscope ZEISS LSM 980 (Carl Zeiss AG, RRID:SCR_025048) equipped with a thermostatic humid chamber with 5% CO_2_ and 37°C. All images were acquired with a 10 × 0.3-NA air objective (model EC Plan-Neofluar 10×/0.30 M27), inverted microscope Axio Observer Z1/7, and LSM980 Airyscan detector 1.3× zoom, laser 488 nm at 0.7% power. Images were acquired every 30 minutes for 18 hours across 20 fields of view per condition per experiment. Raw files were processed in Zen 3.4 Blue software (Carl Zeiss, RRID:SCR_013672) with Airyscan-Processing, the fields of view were stitched, and files were exported with OME-TIFF export. The processed images were analyzed with the TrackMate v7 plugin in Fiji/ImageJ (National Institutes of Health, RRID:SCR_002285; ref. 13). The TrackMate parameters were optimized separately for each cell line. The optimized parameters were used for batch analysis with TrackMate Batcher v1.2.3. Information about migration speed and directionality index was extracted from the tracks’ features calculated in TrackMate. The directionality index was calculated as the net distance (Euclidean distance) between the start and finish divided by the total distance traveled (14).

### Immunohistochemical staining and scoring

Dedifferentiated liposarcoma (DDLPS) tissue microarrays (TMA) were used for immunohistochemical (IHC) assessment of tumor infiltrating lymphocyte (TIL) markers CD3 (DAKO, clone M0452, 1:600), CD4 (DAKO clone 4B12, 1:80), and CD8 (DAKO clone C8/144B, 1:100, RRID:AB_2075537). For validation of mass spectrometry results, the LMS specimens from the proteomics cohort were stained for lymphocyte cytosolic protein 1 (LCP1, Atlas Antibodies, HPA019493, 1:100, RRID:AB_1855457) in a TMA format. In addition, TMAs comprising DDLPS, LMS, and undifferentiated pleomorphic sarcoma (UPS) specimens from the proteomics cohort and an independent cohort were stained for matrix metallopeptidase 14 (MMP14, Sigma-Aldrich, MAB3328, 1:200). Serial 4 μm TMA sections were cut and mounted on slides. The slides were deparaffinized with xylene and rehydrated in a series of ethanol washes with decreasing concentration. DAKO link automated stainer (Agilent) with an EnVision FLEX kit (K8002; Agilent) was used for TIL IHC processing. Antigen retrieval was performed by either pressure cooking in citrate (pH 6) for 2 minutes (CD3) or incubating with pH9 pretreatment module buffer (Agilent) for 20 minutes at 97°C (CD4 and CD8). The slides were stained with relevant primary antibodies for 60 minutes at room temperature. LCP1 and MMP14 staining was done manually. Antigen retrieval was performed in sodium citrate buffer (pH 6) for 10 minutes in a microwave oven and cooled for 45 minutes at room temperature. Sections were blocked with 3% (m/v) bovine serum albumin (Sigma-Aldrich) in Tris-buffered saline-Tween buffer for 90 minutes in a humidity chamber at room temperature. Sections were incubated with relevant primary antibodies in a humidity chamber at 4°C overnight. Secondary antibody staining was performed using mouse or rabbit horseradish peroxidase–linked antibodies and applying 3,3′diaminobenzidine (DAB) substrate. The slides were then counterstained with hematoxylin, dehydrated, and mounted in Pertex mounting medium (Pioneer).

The CD3/4/8^+^ TIL counts were conducted under direct brightfield microscopy at a magnification of ×400. Cell counts for cores with section preservation ranging from 50% to 100% were adjusted to represent 100% of the area. Data from cases with section preservation below 50% were excluded from the analysis. At least two replicate scores were averaged and multiplied by 1.274 to calculate the average CD3^+^, CD4^+^, or CD8^+^ TILs/mm^2^. Digital microscopy images for all stained TMA sections were captured at a resolution of ×40 using the Nanozoomer-XR microscope (Hamamatsu Photonics).

Given intracellular, transmembrane, and extracellular localization of MMP14, QuPath (RRID:SCR_018257) was used for positive pixel counting of MMP14 staining as previously described (15). TMA cores were manually selected so that the entire tissue sample was included. Positive pixels were counted with resolution 0.9 μm/px, Gaussian sigma 1 μm and “positive” DAB threshold 0.15 OD units. Given the cytoplasmic localization of LCP1, LCP1 positive cell detection was performed in QuPath using the requested pixel size of 0 μm, nucleus parameters with a minimum area of 10 μm^2^, maximum area of 400 μm^2^, sigma of 0.6 μm, and threshold of 0.2. Positive LCP1 staining was detected in the cytoplasm (DAB OD mean) with thresholds 0.14 OD for low, 0.18 OD for intermediate, and 0.23 OD for high staining. LCP1 staining was scored using the H-score method (16). For each case, the average value MMP14 or LCP1 score was calculated across two replicate cores selected at random. Gaussian mixture modeling was performed using the mclust R package to fit mean LCP1 scores per patient into three clusters (low, intermediate, and high). The Mann–Whitney *U* test was used to compare LCP1 expression derived from proteomic experiments against IHC H-score-derived LCP1 clusters.

### Statistical analysis

All statistical tests were two-sided. As applicable, data distribution was evaluated for normality using the Shapiro–Wilk test, and in cases where *P* < 0.05, nonparametric tests that do not rely on the assumption of a normal distribution were applied. The statistical tests used included Kruskal–Wallis one-way analysis of variance (ANOVA) test, Dunn’s test, one-way ANOVA test, Tukey’s honestly significant difference (HSD) test, Mann–Whitney *U* test, and χ^2^ test of independence. Details for survival analyses, proteoglycan prognostic score, and The Cancer Genome Atlas (TCGA)-SARC analysis are provided in the Supplementary Methods S1.

### Data availability

Proteomics data from patient specimens were obtained from ProteomeXchange (PXD036226). Cell line and LMS ECM hydrogel proteomics data have been deposited to the ProteomeXchange Consortium via the PRIDE (17) partner repository with the dataset identifiers PXD049373 and PXD049383, respectively. The RNA sequencing raw counts along with the clinicopathological features were downloaded from the public database TCGA Program at the Genomic Data Commons Portal (https://portal.gdc.cancer.gov/, GDC TCGA Sarcoma cohort). Other data generated in this study are available upon request from the corresponding author.

## Results

### Global characterization of matrisome and adhesome profiles in STS

**Figure 1.**
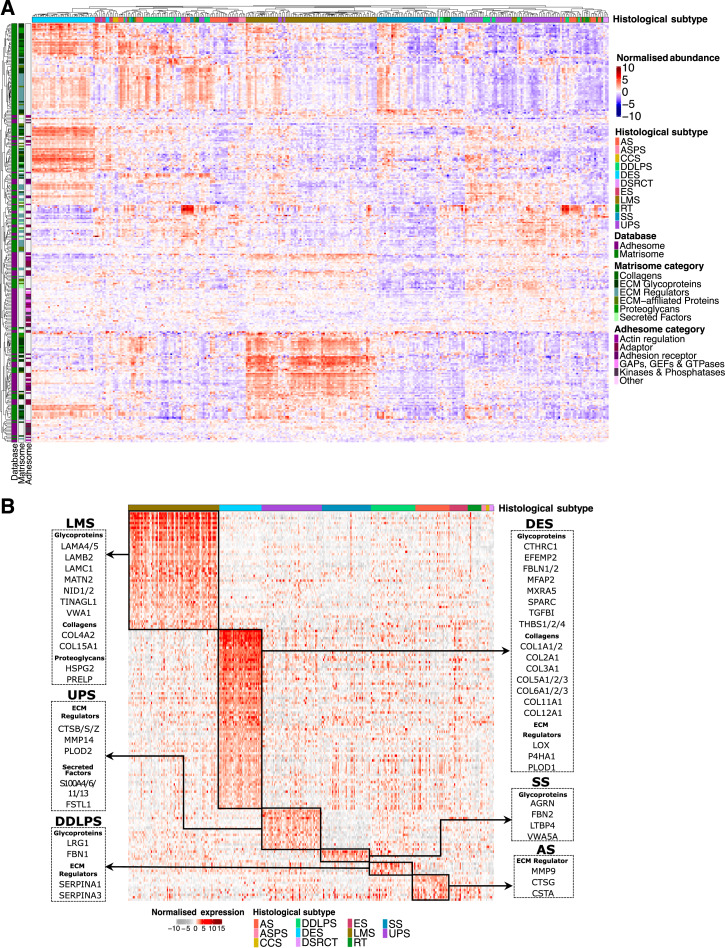
The matrisome and adhesome landscape of soft tissue sarcomas (STS). **A,** Annotated heatmap illustrating the unsupervised clustering (Pearson’s correlation distance) of 302 matrisome and adhesome components in the STS cohort. Top annotation panel correspond to histological subtype. The annotation on the (left) side shows proteins belonging to matrisome or adhesome databases and the breakdown into matrisome and adhesome functional classes. **B,** Heatmap showing matrisome and adhesome proteins uniquely upregulated (indicated by black boxes) in histological subtypes (false discovery rate <0.01, fold change ≥2), arranged by histological subtype. A selection of matrisome proteins which are upregulated in each histological subtype is shown. AS, angiosarcoma; ASPS, alveolar soft part sarcoma; CCS, clear cell sarcoma; DSRCT, desmoplastic small round cell tumor; ES, epithelioid sarcoma; RT, rhabdoid tumor.

The cohort comprises localized primary surgical specimens from 321 patients across 11 histological subtypes for which proteomic profiles were previously acquired by mass spectrometry (8). The baseline clinicopathological variables of the cohort are summarized in Table 1. The matrisome is an *in silico* compilation of all ECM and ECM-associated genes (18). Similarly, the integrin adhesome is a literature-curated database of proteins involved in integrin-mediated adhesion complexes (4, 5). Of the 3,290 proteins quantified across all cases, 193 matrisome proteins were annotated in the MatrisomeDB database (9) and 109 proteins in the integrin adhesome database (Supplementary Table S1; refs. 4, 5). This represents 19% and 47% coverage of the proteins defined in each of the two databases (Supplementary Fig. S1A). The breakdown of proteins in each of the matrisome and adhesome functional classes is outlined in Supplementary Fig. S1B. Unsupervised clustering of the matrisome and adhesome proteins (*n* = 302) showed that STS cases clustered largely by histological subtypes (Fig. 1A) with desmoid tumors (DES) and LMS having the most distinct proteomic profiles. Uniform manifold approximation and projection for dimension reduction analysis confirms this finding and indicates that despite arising from a range of anatomical sites that may represent different stromal microenvironments, STS do not cluster by anatomical location but rather by histological subtype (Supplementary Fig. S1C).

**Table 1.** Clinicopathological characteristics of the cohort.

Total number of patients [*n* (%)]	—	321 (100)
Histological subtype [*n* (%)]	Angiosarcoma	30 (9)
	Alveolar soft part sarcoma	4 (1)
	Clear cell sarcoma	3 (1)
	Dedifferentiated liposarcoma	39 (12)
	Desmoid tumor	37 (12)
	Desmoplastic small round cell tumor	4 (1)
	Epithelioid sarcoma	16 (5)
	Leiomyosarcoma	80 (25)
	Rhabdoid tumor	12 (4)
	Synovial sarcoma	43 (13)
	Undifferentiated pleomorphic sarcoma	53 (17)
Age at excision (years)	Median	58.4
	Min	0.1
	Max	90
Anatomical site [*n* (%)]	Extremity	125 (38.9)
	Head/neck	13 (4.0)
	Intra-abdominal	28 (8.7)
	Retroperitoneal	57 (17.8)
	Trunk	65 (20.2)
	Pelvic	24 (7.5)
	Uterine	9 (2.8)
Grade [*n* (%)]	2	115 (35.8)
	3	139 (43.3)
	*unknown*	67 (20.9)
Tumor depth [*n* (%)]	Deep	250 (77.9)
	Superficial	54 (16.8)
	*unknown*	17 (5.3)
Tumor size (mm)	Median	90
	Min	4
	Max	1,090
Tumor margins [*n* (%)]	R0	133 (41.4)
	R1	151 (47.0)
	R2	4 (1.2)
	*unknown*	33 (10.3)
Pre-op treatment [*n* (%)]	Chemo	19 (5.9)
	Radio	8 (2.5)
	Chemo and radio	13 (4.0)
	None	267 (83.2)
	*unknown*	14 (4.4)
Performance status [*n* (%)]	0	158 (49.2)
	1	82 (25.5)
	2	16 (5.0)
	3	5 (1.6)
	*unknown*	60 (18.7)
Sex [*n* (%)]	F	201 (62.6)
	M	119 (37.1)
	*unknown*	1 (0.3)
Status at excision [*n* (%)]	Local	301 (93.8)
	Metastatic	15 (4.7)
	Locally metastatic	3 (0.9)
	Multifocal	1 (0.3)
	*unknown*	1 (0.3)

We then sought to define the matrisome components that were significantly enriched in each histological subtype (with at least 20 cases; Fig. 1B). LMS have elevated levels of basement membrane proteins including collagens (COL4A2, COL15A1), laminins (LAMA4, LAMA5, LAMB2, LAMC1), and glycoproteins (MATN2, NID1, NID2, TINAGL1, and VWA1; ref. 19). In contrast, DES was enriched in fibrillar collagens (COL1A1/2 COL2A1, COL3A1, COL5A1/2/3, and COL6A1/2/3) and collagen synthesis and remodeling enzymes (P4HA1, PLOD1, LOX), which is consistent with the abundant dense collagenous matrix characteristic of this disease (20). UPS showed upregulation in ECM proteases including the cathepsins (CTSB, CTSS, and CTSZ) and MMP14. In addition, this subtype displayed elevated levels of multiple members of the S100A family of secreted proteins, which are known to play important roles in immune homeostasis (21). Upregulation of MMP14 in UPS was confirmed by IHC staining in our cohort as well as an independent cohort (Supplementary Fig. S2A–S2C). Only a small number of proteins were uniquely upregulated in angiosarcomas, synovial sarcomas (SS), and DDLPS. Collectively our analysis demonstrates that distinct histological subtypes have characteristic ECM profiles which can shed light on the biological features inherent in these diseases.

The proteomic data were generated from cases enriched for >75% tumor content (8). We evaluated if quantitative differences in matrisome proteins as a function of percentage tumor content were observed in our proteomic dataset. Focusing on matrisome proteins that were enriched in desmoid tumors (Fig. 1B), we show that when classified by percentage tumor content (75%–80%, ≥80%–90%, and ≥90%), there was no statistically significant difference between the matrisome protein expression at the global level (Supplementary Fig. S3A—all matrisome proteins) or at the individual protein level across the different matrisome classes (Supplementary Fig. S3B). This analysis provides evidence that the amount of nontumor component (up to 25% nontumor content) in the tissue does not impact the observed ECM results in our proteomic data. We further evaluated if preoperative treatment modulates the matrisome in SS. Analysis of cases that had undergone preoperative treatment versus those that did not finds that 54 matrisome proteins were significantly upregulated upon treatment, particularly in those cases that received neoadjuvant radiotherapy (Supplementary Fig. S4A and S4B). Ontology analysis identifies the complement and coagulation cascade as well as ECM–receptor interactions as key networks that are enriched upon preoperative treatment (Supplementary Fig. S4C and S4D). No proteins were found to be significantly downregulated in the SS cases that received preoperative therapy.

### Coregulated matrisome and adhesome networks are associated with clinicopathological variables

**Figure 2.**
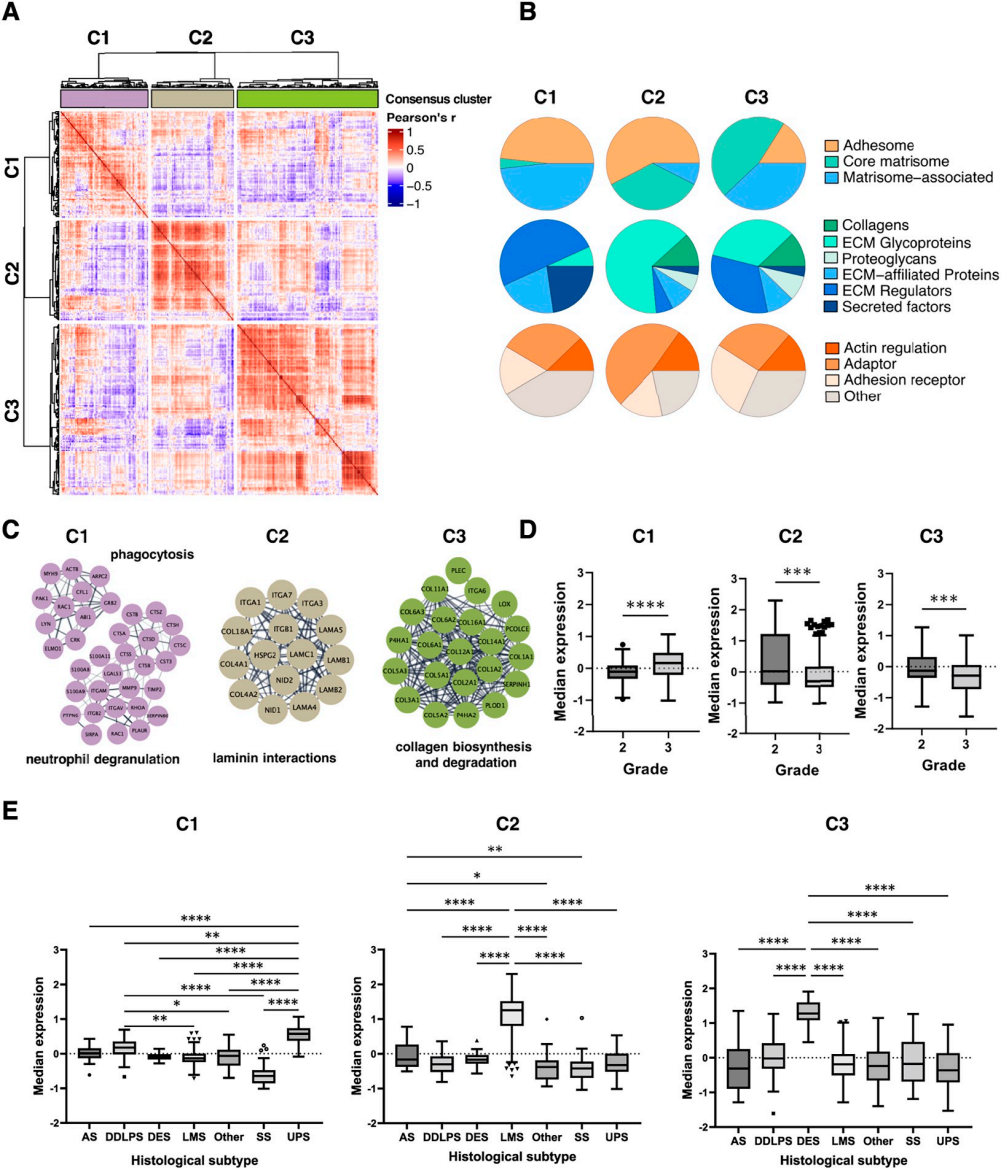
Matrisome and adhesome networks in STS. **A,** Heatmap showing a similarity matrix of Pearson’s correlation coefficients for all pairwise comparisons of matrisome and adhesome proteins. Heatmap is split into three clusters (C1, C2, and C3) identified by consensus clustering analysis. **B,** Pie charts showing breakdown of proteins within the clusters into adhesome, core matrisome, or matrisome-associated proteins (top), breakdown by matrisome class (middle) and by functional annotation of adhesome (bottom). **C,** Selected protein–protein interaction networks (colored by clusters) are shown for each cluster as identified by enrichment analysis (reactome pathways). **D,** Box plots showing distributions of median expression of C1, C2 and C3 proteins across tumor grades. Boxes indicate 25th and 75th percentile, with the median line in the middle, whiskers extending from 25th percentile − [1.5 × interquartile range (IQR)] to 75th percentile + (1.5 × IQR), and outliers plotted as points. Significance determined by Mann–Whitney *U* test. ***, *P* < 0.001; ****, *P* < 0.0001. **E,** Box plots showing distributions of median expression of C1, C2 and C3 proteins across histological subtypes. Boxes indicate 25th and 75th percentile, with the median line in the middle, whiskers extending from 25th percentile − (1.5 × IQR) to 75th percentile + (1.5 × IQR), and outliers plotted as points. Significance determined by Kruskal–Wallis tests with Dunn’s multiple corrections tests. *, *P* < 0.05; **, *P* < 0.01; ****, *P* < 0.0001. Other, ASPS, alveolar soft part sarcoma; CCS, clear cell sarcoma; DSRCT, desmoplastic small round cell tumor; ES, epithelioid sarcoma; RT, rhabdoid tumor.

The matrisome is a complex milieu of multiple proteins that act together as a coordinated network of biochemical and biophysical signals which initiate integrin-mediated adhesome signaling pathways. To identify coregulated ECM signaling networks intrinsic to STS, we undertook Pearson’s correlation analysis of all pairwise comparisons of each of the 302 matrisome/adhesome proteins within the dataset. Consensus clustering identified three distinct clusters of coregulated proteins (C1-3) that were determined to be significant by SigClust (*P* < 0.0001; ref. 22) and are shown in the similarity matrix in Fig. 2A (composition of each cluster provided in Supplementary Table S2).

An assessment of the proteins in each cluster showed that there were distinct proportions of core matrisome, matrisome-associated, and adhesome proteins. C1 (*n* = 85) was composed of 41 adhesome, 41 matrisome-associated, and three core matrisome proteins (Fig. 2B). In contrast, C2 (*n* = 80) had a similar number of adhesome proteins (*n* = 46) as C1 but much higher levels of core matrisome (*n* = 28) compared to matrisome-associated proteins (*n* = 6). C3 (*n* = 137) was mainly composed of matrisome proteins (63 core matrisome and 52 matrisome-associated proteins) with only 22 adhesome proteins. Furthermore, evaluation of the matrisome and adhesome classes as defined by Shao and colleagues (9) and Winograd-Katz and colleagues (5), respectively, finds that C1 consisted primarily of ECM regulators (50%), C2 mostly of glycoproteins (65%), and C3 a mix of both glycoproteins (34%) and ECM regulators (32%). Interestingly, C3 harbored a fibrotic phenotype and contained 14/18 collagen chains detected in the proteomic dataset. The most notable difference in adhesome composition was an enrichment in the adaptor category in C2 (48%) compared to C1 (29%) and C3 (27%). Overrepresentation analysis showed that C1 was significantly enriched for biological processes related to the innate immune system and phagocytosis processes (Supplementary Table S3) with the subnetworks for “neutrophil degranulation” and “Fc gamma receptor dependent phagocytosis” represented in Fig. 2C. C2 was enriched for laminin interactions, while C3 was enriched for collagen biosynthesis and degradation (Supplementary Fig. S2C; Supplementary Table S3).

We then determined if the median expression levels of coregulated proteins in each of the three clusters were associated with the clinicopathological variables of tumor malignancy grade and histological subtype (Fig. 2D and E). When evaluating tumor grade, C1 protein levels were reduced in grade 2 compared to grade 3 tumors (*P* < 0.0001; Fig. 2D). In contrast, proteins in C2 (*P* = 0.0006) and C3 (*P* = 0.0004) showed decreased expression in grade 3 versus grade 2 tumors. Note that for the assessment of tumor grade, DES cases were excluded, as this tumor type is not typically graded. Evaluation of the association with histological subtypes showed that C1 was significantly upregulated in UPS, C2 in LMS, and C3 in DES (Fig. 2E). In addition, there was a significant reduction of C1 protein levels in SS compared to all other histologies (Supplementary Table S4). Our data indicate that the matrisome and adhesome signaling networks in STS can be broadly grouped into three coregulated protein–protein interaction networks which are associated with tumors of distinct histological type and tumor grade.

### Patient-derived ECM modulates cell migration and identifies the LCP1 adhesion protein as a prognostic factor in LMS

**Figure 3.**
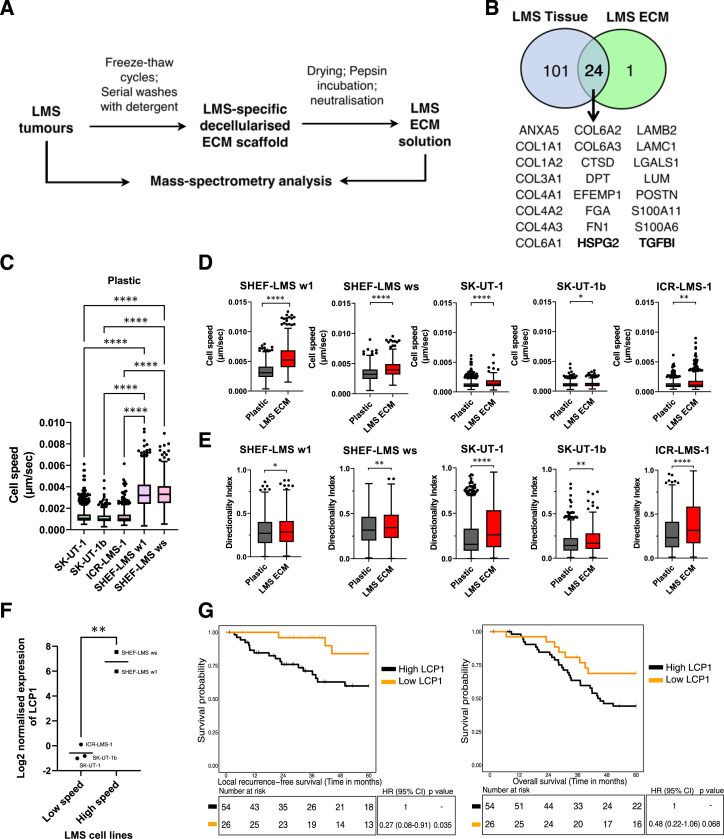
Generation and characterization of LMS ECM solution. **A,** A workflow to generate decellularized ECM LMS scaffolds from fresh frozen LMS tumors by extensive washes with detergent and generation of LMS ECM solution by incubating dried scaffolds with acidified pepsin, followed by neutralization with sodium hydroxide. Paired tumor and solidified LMS ECM solution samples were characterized by mass spectrometry. **B,** Venn diagram showing overlap of matrisome protein IDs consistently detected in all tumors and LMS ECM solution samples. Identities of 24 overlapping proteins are shown at the bottom. **C,** Box plots showing the 2D migration cell speed on plastic in GFP^+^ SK-UT-1 (*n* = 910), SK-UT-1b (*n* = 495), ICR-LMS-1 (*n* = 528), SHEF-LMS w1 (*n* = 996), and SHEF-LMS ws (*n* = 1,024) cells over 18 hours. Significance is shown following Kruskal–Wallis test with Dunn’s multiple testing correction. ****, *P* < 0.0001. **D,** Box plots showing the cell speed of GFP^+^ LMS cell lines on plastic or plastic precoated with LMS ECM solution. Significance is shown following Mann–Whitney *U* test. *, *P* < 0.05; **, *P* < 0.01; ****, *P* < 0.0001. **E,** Box plots showing the directionality indices of GFP^+^ LMS cell lines when grown in 2D conditions on plastic or plastic precoated with LMS ECM solution. Significance is shown following Mann–Whitney *U* test. *, *P* < 0.05; **, *P* < 0.01; ****, *P* < 0.0001. The GFP^+^ cells were tracked for 18 hours. Data were pooled from three independent experiments for all graphs. Boxes indicate the 25th, median, and 75th percentile, with whiskers extending from the 25th percentile − (1.5 × IQR) to the 75th percentile + (1.5 × IQR), and outliers plotted as points. **F,** Dot plot showing log_2_ normalized expression of LCP1 protein in low baseline speed cell lines (SK-UT-1, SK-UT-1B, and ICR-LMS-1), and in high baseline speed cell lines (SHEF-LMS w1 and SHEF-LMS ws). **G,** Kaplan–Meier plot of LRFS (left) and OS (right) with stratification by LCP1 tertiles in *n* = 80 LMS patients. Low LCP1 group contains patients with lower tertile (≤ −0.23 LCP1 expression), while high LCP1 group is made up of intermediate and high tertiles (> −0.23 LCP1). Hazard ratio, 95% CI, and *P*-values were determined by univariate Cox regression with a two-sided Wald test.

Our analysis shows that LMS tumors are enriched in components of basement membrane ECM (Figs. 1B and 2C). To assess if this basement membrane ECM impacts the functional biology of LMS cells, we extracted patient-derived ECM from flash frozen tumor specimens. Tumors from seven patients with LMS of the extremities were subjected to decellularization in detergent. Decellularization is the process of physically and chemically removing the cellular and genetic (DNA/RNA) component of tissues to yield an acellular ECM scaffold. The scaffold was then pepsin digested and a patient-derived LMS ECM solution was extracted (Fig. 3A). Mass spectrometry analysis of both LMS tumors and extracted ECM solution was performed to determine their matrisome composition. Across the seven patient specimens, 125 and 25 matrisome proteins were identified in the tumors and extracted ECM solution, respectively, with an overlap of 24 shared proteins (Fig. 3B; Supplementary Fig. S5A). These 24 proteins comprise canonical components of the basement membrane, including COL4A1/2, COL6A1/2/3, LAMB2, LAMC1, and FN1 (Fig. 3B; ref. 19), which further confirms the proteomic results previously obtained from FFPE tissue (Fig. 1B). A breakdown of the proteins identified across the six matrisome classes showed that, as previously reported (23, 24), the decellularization and pepsin digestion process retained many of the collagens and proteoglycans in the patient-derived ECM but led to a reduction in glycoproteins and matrisome-associated proteins when compared with the originating tumors (Supplementary Fig. S5A).

We next investigated if the extracted patient-derived ECM influenced LMS cell migration. We evaluated five cell lines derived from high-grade LMS tumors, two which were commercially available (SKUT-1 and SKUT-1B), and three which are low passage patient-derived or xenograft-derived cell lines (SHEF-LMS w1, SHEF-LMS ws, and ICR-LMS-1; ref. 12). Analysis of baseline cell migration on plastic with live cell imaging with time lapse microscopy showed that the SHEF-LMS w1 and SHEF-LMS ws cell lines had significantly higher cell migration speed compared to the other three cell lines (Fig. 3C). When plated on patient-derived ECM, all the five cell lines showed a statistically significant increase in cell migration speed (Fig. 3D), even though the cell lines with low baseline speed (SKUT-1, SKUT-1B, and ICR-LMS-1) had numerically small increases in the presence of patient-derived ECM. When directionality index was assessed, all cell lines showed a statistically significant increase in persistent displacement when plated on LMS ECM compared to plastic (Fig. 3E), although the increase in SHEF-LMS w1, SHEF-LMS ws, and SKUT-1B was numerically small (Supplementary Fig. S5B). Our data indicate that across different LMS cell lines, the addition of patient-derived ECM induced a statistically significant increase in cell migration speed and persistent directional migration, which have previously been shown to be essential processes in promoting cancer progression (25). We further compared the migration properties of cells grown on patient-derived ECM with purified fibronectin, laminin, and collagen IV. When assessing cell speed, 4/5 cell lines (SHEF-LMS w1, SHEF-LMS ws, SK-UT-1, and SK-UT-1b) displayed significantly higher speed on fibronectin compared to LMS ECM, while 3/5 cell lines (SHEF-LMS w1, SK-UT-1b, and ICR-LMS-1) showed higher speed when plated on laminin compared to LMS ECM (Supplementary Fig. S5C). In terms of directionality index, 4/5 cell lines (SHEF-LMS w1, SHEF-LMS ws, SK-UT-1, and ICR-LMS-1) that were grown on LMS ECM had similar properties as fibronectin and collagen IV but this significantly decreased when cells were grown on laminin. In contrast, SK-UT-1b showed significantly higher directionality index when grown on fibronectin compared to LMS-derived ECM (Supplementary Fig. S5D).

To determine if the molecular features driving cell migration in LMS cell lines are associated with patient survival outcomes, we undertook a comparative proteomic analysis of LMS patients and the panel of LMS cell lines. First, we performed a MS-based proteomic analysis of the five LMS cell lines in our panel. Across the panel, 4,160 proteins were identified and quantified (Supplementary Fig. S6A). Significance analysis of microarray (SAM) was used to identify proteins that were associated with high baseline cell migration speed (SHEF-LMS w1 and SHEF-LMS ws) versus low speed (SKUT-1, SKUT-1b, and ICR-LMS-1). This analysis identified four proteins which were significantly different between the two groups (LCP1, MT1L, GSPT2, and FRMPD1). Of these four proteins, only LCP1 was expressed in the proteomic datasets of both LMS patient specimens in our STS cohort (*n* = 80) and the cell lines. LCP1 is a cytoskeletal protein that has been shown to directly interact with multiple integrins and is important in cell adhesion and migration in cancer cells (26, 27). In our analysis, LCP1 shows significantly lower protein expression levels in LMS cells exhibiting low cell migration speed compared to those with high migration speeds (Fig. 3F). In line with this preclinical finding, when interrogating the proteomic data of patient specimens, LMS patients with low LCP1 protein levels (lowest tertile) had superior local recurrence-free survival (LRFS; *P* = 0.035) and overall survival (OS; *P* = 0.068) compared to those with higher protein levels (Fig. 3G). These findings were independently confirmed by IHC staining of LCP1 (Supplementary Fig. S6B–S6E). After adjusting for clinicopathological factors including tumor grade, tumor size, tumor depth, anatomical location, and performance status, LCP1 expression remained an independent prognostic factor for OS and LRFS in the multivariable Cox regression analysis (Supplementary Table S5) [OS: Hazard Ratio (HR) = 0.39; 95% confidence interval (CI) = 0.17–0.91; *P* = 0.029; LRFS: HR, 0.21; 95% CI, 0.06–0.77; *P* = 0.019]. No significant difference was observed in metastasis-free survival (MFS; Supplementary Fig. S6F). Our data highlight a potential utility of LCP1 as a prognostic factor for local recurrence and OS in LMS.

### Matrix remodeling defines DDLPS molecular subgroups with distinct signaling pathways and survival outcomes

DDLPS is an aggressive tumor type of adipocytic origin and accounts for ∼6% of newly diagnosed STS (28). This disease is characterized by clinical heterogeneity in patient outcomes and treatment responses. While historical reports have identified copy number aberrations that define DDLPS subgroups with different survival outcomes (29, 30), these studies lacked the molecular resolution to identify the intrinsic biological pathways within each of these subgroups. Here we sought to determine if ECM remodeling, a process which has previously been shown to fuel aggressive tumor phenotypes in other cancer types (31), could account for the heterogeneity observed in this disease.

**Figure 4.**
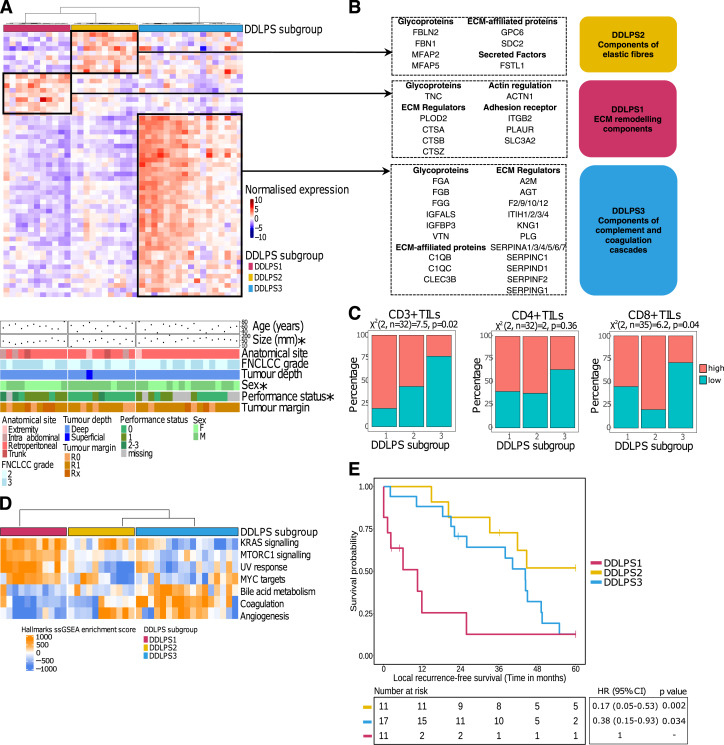
Identification, biological and clinical characterization of DDLPS subgroups. **A,** Heatmap showing the supervised clustering of 57 differentially expressed matrisome and adhesome proteins (DEPs) uniquely upregulated in each DDLPS subgroup. Black boxes indicate unique upregulated matrisome and adhesome DEPs in each of the subgroups. Bottom annotations indicate key tumor and patient characteristics. “*” Indicates that a clinical feature is significantly associated with DDLPS subgroups. **B,** Identities of upregulated matrisome and adhesome proteins in each DDLPS subgroup are shown on the right. Colored boxes on the right show functional pathways enriched in each DDLPS subgroup, as determined by overrepresentation analysis against the reactome pathway database. **(C)** Stacked bar charts showing the percentages of high and low CD3^+^, CD4^+^, and CD8^+^ TIL groups in each DDLPS subgroup. DDLPS cases were divided into high and low categories according to the median TILs score, separately for each stain. The χ^2^ test results are presented at the top of each plot. **D,** Significant (one-way ANOVA; FDR < 0.05) biological features obtained from ssGSEA of the MSigDB Hallmark gene sets. **E,** Kaplan–Meier plot of LRFS with stratification by DDLPS subgroups. Hazard ratio, 95% CI, and *P*-values were determined by univariate Cox regression with a two-sided Wald test.

Consensus clustering of the matrisome/adhesome proteomic profiles in our cohort of 39 DDLPS cases was performed (baseline clinicopathological features summarized in Supplementary Table S6), with three consensus clusters identified (DDLPS1-3 subgroups). A heatmap of the significantly upregulated proteins in each of the three subgroups is shown in Fig. 4A. Among the baseline clinicopathological variables evaluated, the three subgroups were significantly associated with tumor size, sex and performance status (Supplementary Table S7). DDLPS1 was particularly interesting, as it is characterized by elevated levels of ECM remodeling proteins including the cathepsins (CTSA, CTSB, and CTSZ), lysyl hydroxylase (PLOD2) and the plasminogen activator urokinase receptor (PLAUR; Fig. 4B). Matrisome proteins that were upregulated in DDLPS3 comprised two major classes: serine protease inhibitors (SERPIN and ITI family members) and components of the coagulation and complement cascades (Fig. 4B). In line with the reported immune suppressive effects of the complement cascade (32), DDLPS3 harbored an “immune cold” phenotype with significantly reduced levels of CD3^+^ (*P* = 0.024) and CD8^+^ (*P* = 0.044) TILs compared to the other two clusters (Fig. 4C). We also performed single-sample gene set enrichment analysis (ssGSEA) of the full proteomic dataset (*n* = 3,304 proteins) to identify hallmark biological pathways that were intrinsic to each DDLPS subgroup. Compared to DDLPS2 and DDLPS3, DDLPS1 also had high levels of oncogenic signaling such as KRAS signaling (DDLPS2 *P* = 0.0489; DDLPS3 *P* < 0.0001), mTORC1 signaling (DDLPS2 *P* = 0.0003; DDLPS3 *P* < 0.0001) and MYC targets (DDLPS2 *P* = 0.0339; DDLPS3 *P* = 0.0132; Fig. 4D). While there were no significantly enriched ssGSEA hallmarks in DDLPS2, cases in this cluster harbored elevated levels of matrisome proteins involved in elastic fiber formation (FBN1, FBLN2, MFAP2, and MFAP5; Fig. 4B).

Stratifying patients based on the three DDLPS subgroups showed that patients in the DDLPS1 subgroup had significantly inferior 5-year survival outcomes in univariable Cox analysis for LRFS, MFS, and OS compared to DDLPS2 and DDLPS3 (Fig. 4E; Supplementary Fig. S7A and S7B; Supplementary Table S8). Adjusting for clinicopathological variables including age, tumor size, grade, performance status, and sex, the DDLPS1 subgroup remained an independent prognostic factor for LRFS and OS in the multivariable Cox regression analysis (Supplementary Table S9). Altogether, our findings demonstrate that DDLPS is a heterogeneous disease that can be classified based on ECM remodeling features into three subgroups with distinct biological features and survival outcomes.

### A proteoglycan score has prognostic value in UPS and DDLPS

Several groups have previously developed gene expression–based matrix scores for predicting survival outcomes in carcinomas (6, 7). To date, no matrix-based prognostic signatures have been identified for mesenchymal tumors. Focusing on DDLPS and UPS, subtypes that we have previously shown to have elevated levels of the matrisomal components of the complement pathway (8), here we sought to define a prognostic matrix score for these two subtypes (*n* = 92; Supplementary Table S10).

**Figure 5.**
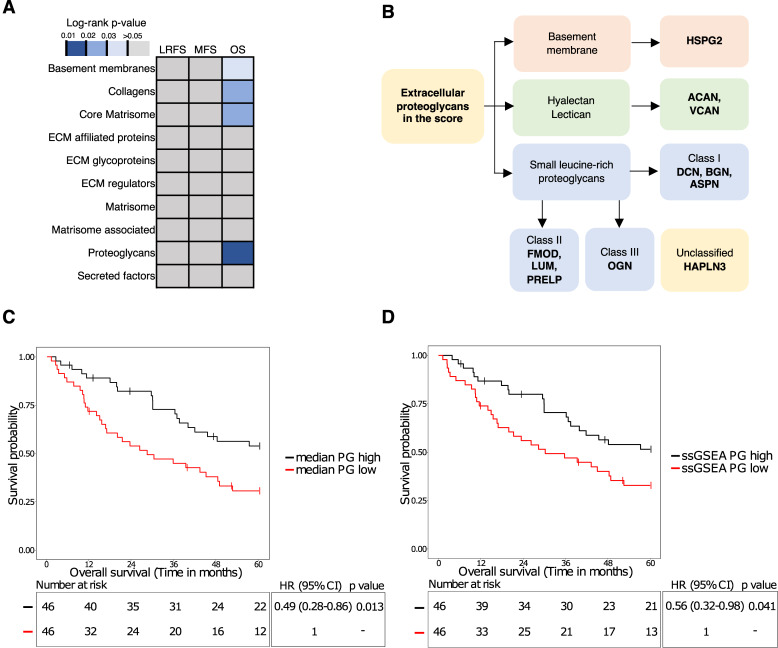
Proteoglycan protein expression identifies a high-risk STS group. **A,** Summary of log-rank tests used to assess significant associations of matrisome-related gene sets with LRFS, MFS, and OS. The scores for each patient were obtained by taking the median expression focusing on 10 matrisome-related gene sets from the Molecular Signatures Database. **B,** Identities of the 11 proteoglycans included in the proteoglycan score. **C,** Kaplan–Meier plot of OS with stratification by the median expression of 11 proteoglycans in a combined UPS and DDLPS cohort (*n* = 92). **D,** Kaplan–Meier plot of OS with stratification by the ssGSEA score of 11 proteoglycans in a combined UPS and DDLPS cohort (*n* = 92). Hazard ratio, 95% CI, and *P*-values were determined by univariate Cox regression with a two-sided Wald test.

To define a matrix score, we started with 10 annotated gene sets comprising different ECM components in MSigDB (Fig. 5A; ref. 18). Median protein expression levels for each ECM gene set were calculated to generate patient-specific median scores. To evaluate the association of these ECM gene set median scores with survival, patients were stratified into high and low groups based on median protein expression levels and log-rank test was used to filter for significant (*P* < 0.05) associations for each of the three survival outcomes (LRFS, MFS, and OS). Of the 10 gene sets, the proteoglycan (PG; *P* = 0.013), basement membrane (*P* = 0.030), collagen (*P* = 0.026), and core matrisome (*P* = 0.024) gene sets were prognostic for OS (Fig. 5A). As multivariable Cox analysis of the basement membrane, collagen, and core matrisome gene sets showed that they were not independent of baseline clinicopathological variables, they were not pursued further (data not shown).

The PG gene set is composed of 11 proteins (Fig. 5B). In univariate Cox regression analysis, patients with a low PG median score had a significantly worse OS compared to patients with a high PG median score (HR, 0.49; 95% CI, 0.28–0.86; *P* = 0.013; Fig. 5C). Additionally, tumor grade, performance status, sex, and histological subtype were significantly associated with the PG score (Supplementary Table S11) with the PG-low subgroup having a higher proportion of grade 3 tumors (*P* < 0.0001), a lower proportion of performance status of 0 (*P* = 0.019), a higher proportion of female (*P* < 0.0001), and a higher proportion of UPS patients (*P* = 0.006). After adjusting for clinicopathological variables of age, tumor grade, histological subtype, anatomical location, tumor size, tumor depth, margins, sex, and performance status, the PG gene set remained an independent prognostic factor (HR, 0.41; 95% CI, 0.19–0.89; *P* = 0.024 (details of univariable and multivariable Cox analysis are provided in Supplementary Table S12). We further assessed the PG gene set using an orthogonal approach with patient-specific ssGSEA scores. Similar to the PG median scores, patients with low PG ssGSEA scores had a significantly worse OS compared to patients with high PG ssGSEA scores (HR, 0.56; 95% CI, 0.32–0.98; *P* = 0.041) in univariate Cox regression analysis (Fig. 5D). After adjusting for confounding clinicopathological variables, the PG ssGSEA score did not remain an independent prognostic factor (HR, 0.49; 95% CI, 0.23–1.02; *P* = 0.058; details of univariable and multivariable Cox analysis are provided in Supplementary Table S13). The consistency of our results using two different scoring methods (median score and ssGSEA score) highlights the robust prognostic value of the PG set of 11 proteins.

We then sought to validate our findings using the gene expression data from DDLPS and UPS cases within the independent TCGA-SARC cohort (10). The TCGA-SARC cohort comprises 91 patients (DDLPS, *n* = 47; UPS, *n* = 44). When stratified by PG median scores, patients in the top quartile showed a superior disease-specific survival (DSS) compared to the rest of the patients in the cohort (HR, 0.19; 95% CI, 0.05–0.81; *P* = 0.025; Supplementary Fig. S8A). A similar trend was observed in OS, where patients in the top quartile showed better outcomes versus the rest of the cohort but this was not statistically significant (Supplementary Fig. S8B). Using ssGSEA scores, both DSS and OS were not statistically significant (Supplementary Fig. S8C and S8D), although DSS showed the expected trend of superior survival in patients with high PG ssGSEA scores (Supplementary Fig. S8C).

## Discussion

ECM-mediated bidirectional signaling plays an important role in driving multiple stages of cancer development. Given that the majority of published studies have focused on carcinomas, it remains unknown if mesenchymal tumors possess unique tumor ecosystems with distinct ECM profiles that could be exploited for therapy and biomarker discovery (2). Here, we provide a comprehensive proteomic map of ECM and integrin adhesion networks in a broad range of STS subtypes. We further perform subtype-specific analyses in LMS, DDLPS, and UPS because these are some of the most common STS histologies. To our knowledge, this is the first detailed characterization of the ECM composition in STS, which has shed light on the key matrix remodeling events operating in this group of diseases, with demonstrated applications in molecular-based disease classification and patient risk stratification.

One of the key findings of our study is the considerable ECM heterogeneity in STS, with several histologies having subtype-specific unique ECM profiles. For instance, LMS primarily contain basement membrane enriched ECMs while DES have high levels of fibrillar collagens and collagen-modifying enzymes. Our analysis of coregulated signaling networks was also able to reveal important biological features in subtypes such as UPS where leukocyte infiltration, neutrophil degranulation, and phagocytosis proteins were significantly upregulated, a finding which is consistent with prior studies demonstrating that UPS is one of the most “immune hot” tumors across STS subtypes (33). Our results can be exploited to rationally select next-generation antistromal therapies for personalized treatment. For example, bicyclic peptides that bind to MMP14 have been employed as delivery systems for imaging agents and drug payloads (34). BT1718 is a bicyclic peptide conjugated to mertansine that is currently being evaluated in a phase I/II clinical trial in solid tumors including sarcomas (NCT02386730). Our data suggest that to improve the likelihood of success, rather than a “one size fits all” pan-sarcoma approach, future trials evaluating this agent should be selectively enriched for the UPS subtype which harbors particularly high levels of MMP14.

The bulk of preclinical studies, including large-scale cancer dependency maps and drug sensitization screens, have relied on cancer cells grown on plastic that do not faithfully recapitulate ECM biochemical properties and heterogeneity that we have shown to be present in STS tumors. This lack of a representative ECM component may be one reason why a large proportion of candidate therapies identified with conventional preclinical models fail to translate into positive clinical trials. The use of well-characterized patient-derived ECM models which capture clinically relevant subtype-specific matrix components may increase translational success. Here we show that ECM extracted from multiple LMS patients share a conserved set of basement membrane proteins that is reflective of the originating tumor specimens. We further demonstrate that compared to plastic, patient-derived ECM has an impact on LMS cellular phenotypes such as cell migration and directional persistence, although some of the differences are numerically small. Our findings illustrate the importance of incorporating these critical elements of the sarcoma TME into future preclinical studies in STS. Notably, our study also highlights the potential of *in vitro* cell migration screens as a novel approach for identifying prognostic factors for disease outcomes. We show that the cytoskeletal adhesion protein LCP1 is decreased in LMS cell lines with low migration speeds and low protein expression is associated with better OS and LRFS outcomes in LMS patients. Consistent with our findings, LCP1 has been found to promote cell migration and invasion in osteosarcoma (35) and chondrosarcoma (bioRxiv 2023:2023.01.31.526513) cell lines. In particular low *LCP1* mRNA levels are associated with significantly better OS in chondrosarcoma patients (bioRxiv 2023:2023.01.31.526513). Collectively, these data propose LCP1 as a candidate driver of cancer progression in sarcomas and a target for future drug development.

DDLPS is an aggressive disease where high-risk patients are prone to relapse following surgery of localized disease with curative intent (28). While the genomic drivers of this disease are relatively well characterized (e.g., *CDK4* and *MDM2* amplification), clinical trials evaluating targeted agents to these drivers have reported disappointing results (36, 37). This suggests that there are additional epigenetic or proteomic features that contribute to disease heterogeneity. Here we show that DDLPS can be categorized into three molecular subgroups based on differences in matrisome and adhesome profiles. In particular, the DDLPS1 high-risk subgroup harbored high levels of ECM remodeling enzymes including PLOD2 and PLAUR. Consistent with their poor survival outcomes, these patients also had the highest levels of oncogenic signaling mediated by mTORC1 and MYC. PLOD2 has been reported to promote metastasis is a mouse model of UPS and use of the PLOD2 inhibitor minoxidil inhibits cell migration and *in vivo* pulmonary metastasis (38). *PLAUR,* which encodes for uPAR, has been shown to drive tumor proliferation and metastasis in multiple cancer types including pediatric rhabdomyosarcoma and osteosarcoma (39, 40). Recent advances in the development of immunotherapies targeted against uPAR, such as anti-uPAR chimeric antigen receptor (CAR) T cells and antibody recruiting molecules, show promising durable preclinical responses (41, 42). Taken together, our study nominates several key antistromal therapeutic options for high-risk DDLPS patients which may complement CDK4- and MDM2-targeted inhibitors that have thus far had limited efficacy in clinical trials.

We have identified a PG score that is associated with superior 5-year OS in patients with UPS and DDLPS. This score comprises 11 proteins across different PG classes including the small leucine-rich PG Class I (BGN, DCN, ASPN), Class II (FMOD, LUM, PRELP), and Class III (OGN) proteins (43). It also includes PGs of the hyalectan/lectican class (ACAN, VCAN) and basement membrane zone (HSPG2). PGs have been shown to play both protumorigenic and tumor suppressor functions in different cancer types. Our data indicate that in the context of STS, these proteins are likely to have a tumor-suppressive role. Indeed, a number of proteins within the PG score, such as LUM, HSPG2, DCN and BGN, have established tumor suppression functions in other cancer types (44–47). This PG score has potential utility in the prospective identification of high-risk patients to inform clinical management. including consideration for more intensive treatment regimens such as perioperative therapy. When validating our findings using the transcriptomics dataset from the independent TCGA-SARC cohort, we showed that while there was no association with OS, patients with high PG median scores had superior DSS compared to the rest of the cohort. While our findings are promising, it should be noted that our study is a retrospective analysis that is susceptible to selection bias and our biomarker findings should be considered hypothesis generating with a need for future validation in larger independent cohorts.

There are several limitations of our study. Due to the nature of bulk proteomic analysis, it is not possible to precisely identify the cell of origin or spatial localization of the ECM and adhesome components in our study. This information will be important to better understand the mechanistic roles of ECM remodeling in sarcomas. It is anticipated that exciting developments in single cell and spatial proteomic approaches will complement our proteomic resource in future studies. Unlike other Omics approaches such as transcriptomics, it is still not possible to achieve genome-wide coverage with routine shotgun mass spectrometry–based proteomics. As such we were only able to characterize a subset of the proteins within the MatrisomeDB and adhesome databases, which could result in a potential bias in downstream data analysis. As the ECM mediates its effects predominantly at the protein rather than the transcript level, and considering previous studies that have shown a poor correlation between protein and RNA expression levels (48), in our view, it is critical (in spite of its limited coverage) to define the key matrisome and adhesome at the protein level (49). Finally, while our preclinical data demonstrate the importance of incorporating patient-relevant ECM when undertaking cell biology experiments, the exact functional roles of specific ECM and integrin signaling components on STS cellular phenotypes remain to be investigated. Future functional studies to dissect the contribution of ECM remodeling and integrin signaling to therapy response and cell migration in STS are warranted.

In summary, we have undertaken a comprehensive analysis of the ECM networks operating in STS, which has led to the identification of candidate antistromal therapies for specific subtypes and new prognostication tools. Furthermore, our data provide a catalog of key ECM components present in each histological subtype, which may aid future preclinical modeling efforts of this complex group of diseases. By bridging the gap in the knowledge of role of the ECM in STS, our study provides a unique resource for the sarcoma research community to drive future research in this understudied area.

## Supplementary Material

Supplementary Figure S1 Supplementary Figure S1. The matrisome and adhesome profiles in soft tissue sarcoma (STS).

Supplementary Figure S2 Supplementary Figure S2. Validation of MMP14 expression in undifferentiated pleomorphic sarcoma (UPS), dedifferentiated liposarcoma (DDLPS) and leiomyosarcoma (LMS) by immunohistochemistry (IHC).

Supplementary Figure S3 Supplementary Figure S3. Expression of matrisome proteins in a desmoid tumour cohort (n=37) split into 3 groups based on the % tumour content.

Supplementary Figure S4 Supplementary Figure S4. Differentially expressed matrisome proteins in synovial sarcoma (SS) cases (n=42) that either received preoperative treatment or were untreated.

Supplementary Figure S5 Supplementary Figure S5. Assessment of leiomyosarcoma (LMS) extracellular matrix (ECM) solution and its effects of cell migration.

Supplementary Figure S6 Supplementary Figure S6. Identification of LCP1 as a candidate prognostic factor in LMS.

Supplementary Figure S7 Supplementary Figure S7. Clinical characterisation of DDLPS subgroups.

Supplementary Figure S8 Supplementary Figure S8. Association of the proteoglycan gene expression score with survival outcomes in The Cancer Genome Atlas sarcoma (TCGA- SARC) cohort.

Supplementary Methods S1 Supplemental Methods

Supplementary Table S1 Supplementary Table S1: Matrisome and adhesome proteins identified across the soft tissue sarcoma (STS) cohort. Table showing annotation of proteins according to matrisome and integrin adhesome databases and the breakdown into functional matrisome and adhesome categories.

Supplementary Table S2 Supplementary Table S2: Matrisome and adhesome cluster membership.

Supplementary Table S3 Supplementary Table S3: Reactome enriched pathways in soft tissue sarcoma (STS) matrisome and adhesome clusters. Tables for each cluster showing enriched term description and false discovery rate (FDR). Top 10 hits with FDR < 0.001 are shown for each cluster.

Supplementary Table S4 Supplementary Table S4: Statistical comparisons between histological subtypes in cluster 1 (C1).

Supplementary Table S5 Supplementary Table S5. Summary of multivariable (MVA) analyses assessing association of clinicopathological factors and LCP1 tertiles with local recurrence-free survival (LRFS) and overall survival (OS). Clinicopathological factors which were significantly associated with survival in univariable analyses were included in the MVA model.

Supplementary Table S6 Supplementary Table S6: Clinicopathological characteristics of n=39 dedifferentiated liposarcoma (DDLPS). Summary features of the cohort. For continuous variables, the median, minimum (min) and maximum (max) values are indicated. For categorical variables, count and percentage are shown.

Supplementary Table S7 Supplementary Table S7: Summary of statistical tests to assess the association between clinicopathological features and dedifferentiated liposarcoma (DDLPS) subgroups.

Supplementary Table S8 Supplementary Table S8: Summary of univariable (UVA) Cox regression analyses assessing the association of clinicopathological factors and dedifferentiated liposarcoma (DDLPS) subgroups with local recurrence-free survival (LRFS), overall survival (OS) and metastasis-free survival (MFS).

Supplementary Table S9 Supplementary Table S9: Summary of multivariable (MVA) Cox regression analyses assessing the association of clinicopathological factors and dedifferentiated liposarcoma (DDLPS) subgroups with local recurrence-free survival (LRFS), overall survival (OS) and metastasis-free survival (MFS).

Supplementary Table S10 Supplementary Table S10. Clinicopathological characteristics of n=92 undifferentiated pleomorphic sarcoma (UPS) and dedifferentiated liposarcoma (DDLPS) cases. Summary features of the cohort. For continuous variables, the median, minimum (min) and maximum (max) values are indicated. For categorical variables, count and percentage are shown.

Supplementary Table S11 Supplementary Table S11. Statistical association of clinicopathological features with proteoglycan groups.

Supplementary Table S12 Supplementary Table S12. Summary of univariable (UVA) and multivariable (MVA) Cox regression analyses assessing the association of clinicopathological factors and median proteoglycan score with overall survival (OS).

Supplementary Table S13 Supplementary Table S13. Summary of univariable (UVA) and multivariable (MVA) Cox regression analyses assessing the association of clinicopathological factors and ssGSEA-derived proteoglycan score with overall survival (OS).
