# Supplementary material for: Clinical Implications and Molecular Features of Extracellular Matrix Networks in Soft Tissue Sarcomas
Source: Clin Cancer Res. 2024 May 29;30(15):3229–42. doi: 10.1158/1078-0432.CCR-23-3960 (PMC11292195; doi:10.1158/1078-0432.CCR-23-3960)
Supplement: Supplementary Figure S1 — The matrisome and adhesome profiles in soft tissue sarcoma (STS). [file ccr-23-3960_supplementary_figure_s1_suppsf1.pdf]

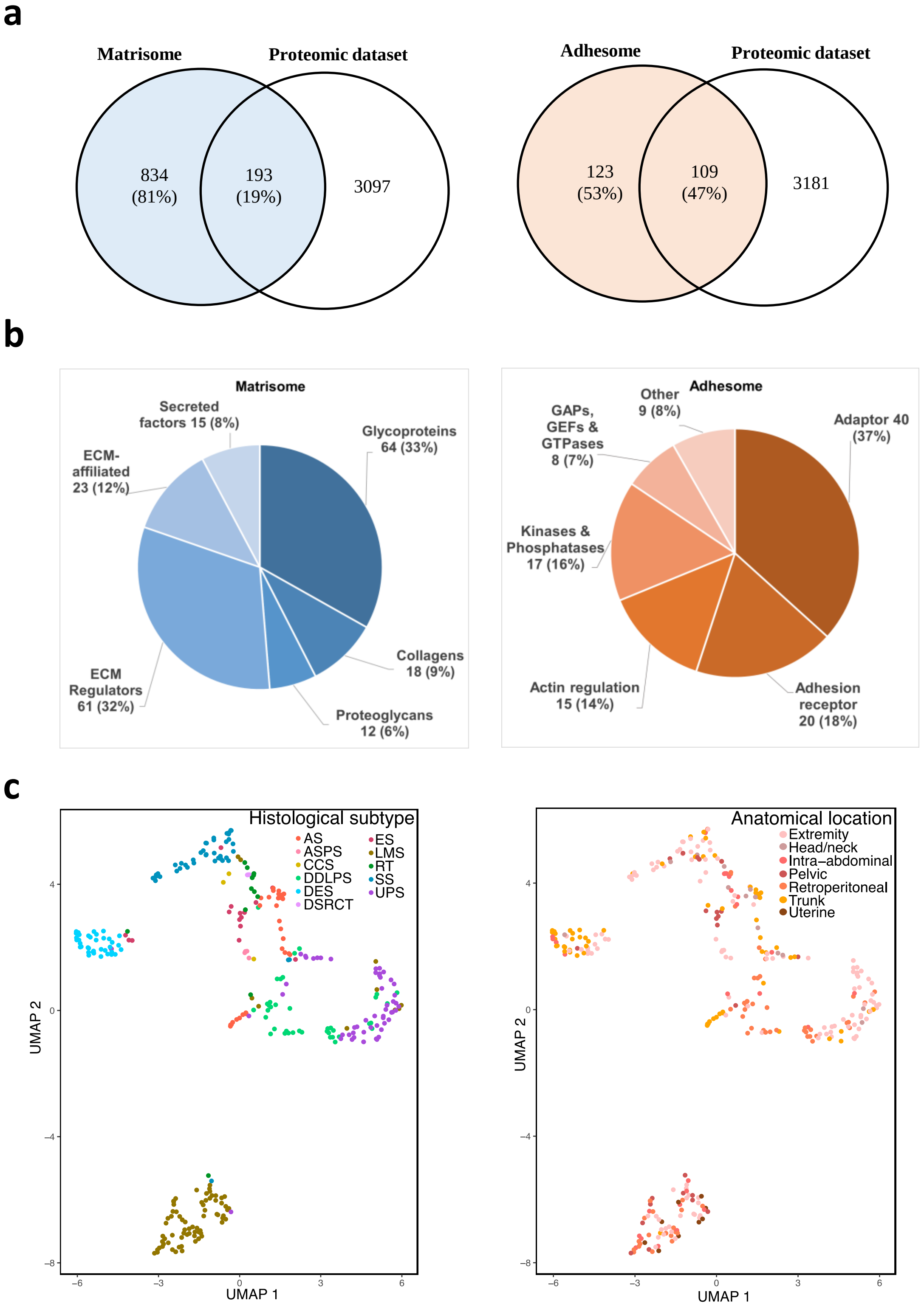

Supplementary Figure S1. The matrisome and adhesome profiles in soft tissue sarcoma (STS). (a) Venn diagrams to show the overlap between the proteomic dataset and matrisome database (left) and overlap between the proteomic dataset and adhesome database (right). (b) Pie charts showing the breakdown of proteins in each of the matrisome (left) and adhesome (right) functional classes. (c) Uniform Manifold Approximation and Projection (UMAP) plots showing matrisome and adhesome proteomic expression of individual patient cases coloured by histological subtype (left) and coloured by anatomical location (right). AS: angiosarcoma, ASPS: alveolar soft part sarcoma, CCS: clear cell sarcoma, DDLPS: dedifferentiated liposarcoma, DES: desmoid sarcoma, DSRCT: desmoplastic small round cell tumor, ECM: extracellular matrix, ES: epitheliod sarcoma, GAP: GTPase-activating protein, GEF: guanine nucleotide exchange factor, GTPase: guanosine triphosphatase, LMS: leiomyosarcoma, RT: rhabdoid tumour, SS: synovial sarcoma, UPS: undifferentiated pleomorphic sarcoma.
