# Supplementary material for: Clinical Implications and Molecular Features of Extracellular Matrix Networks in Soft Tissue Sarcomas
Source: Clin Cancer Res. 2024 May 29;30(15):3229–42. doi: 10.1158/1078-0432.CCR-23-3960 (PMC11292195; doi:10.1158/1078-0432.CCR-23-3960)
Supplement: Supplementary Figure S2 — Validation of MMP14 expression in undifferentiated pleomorphic sarcoma (UPS), dedifferentiated liposarcoma (DDLPS) and leiomyosarcoma (LMS) by immunohistochemistry (IHC). [file ccr-23-3960_supplementary_figure_s2_suppsf2.pdf]

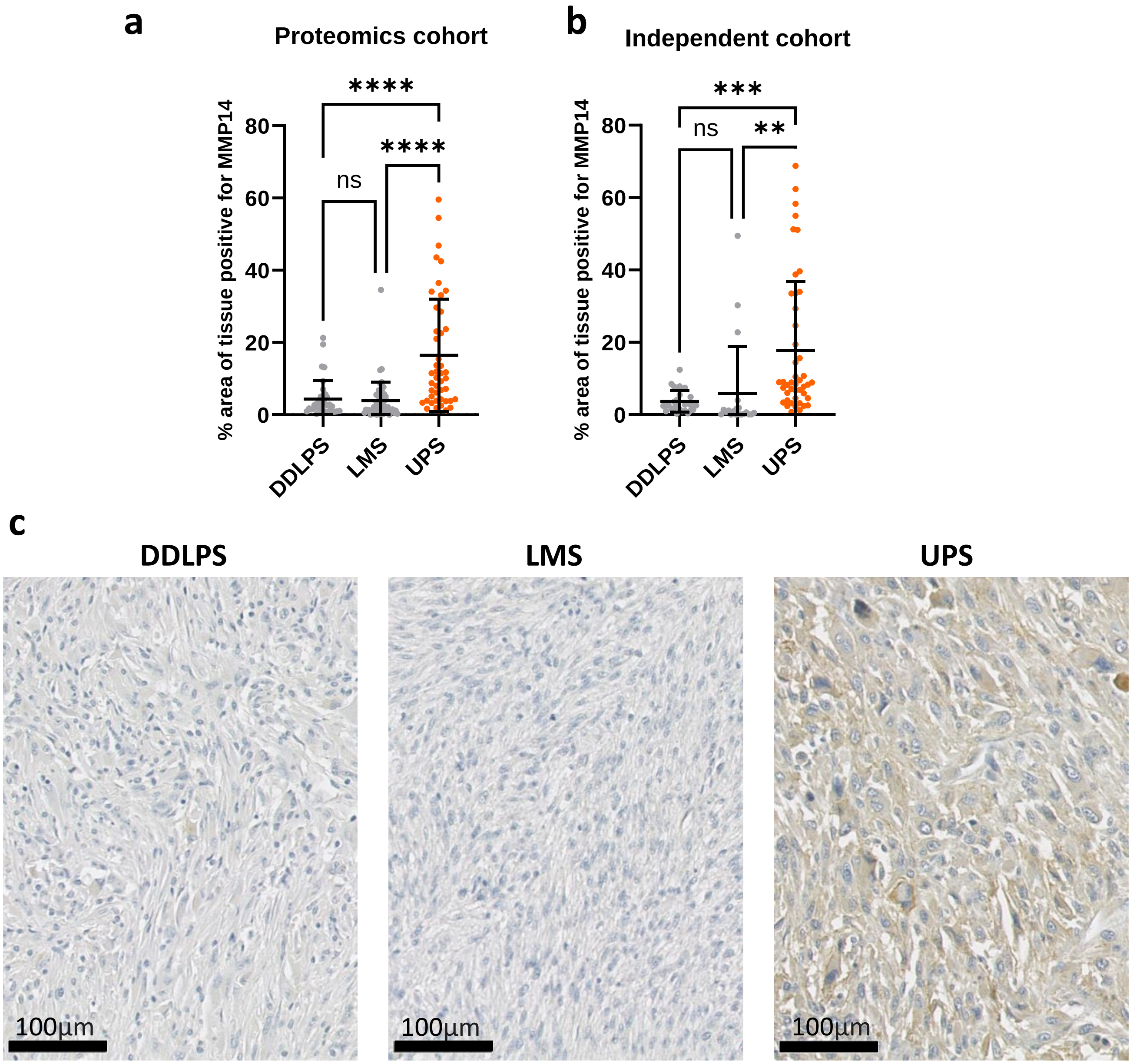

Supplementary Figure S2. **Validation of MMP14 expression in undifferentiated pleomorphic sarcoma (UPS), dedifferentiated liposarcoma (DDLPS) and leiomyosarcoma (LMS) by immunohistochemistry (IHC).** (a) Quantification of percentage of the area of the tissue positive for MMP14 in DDLPS (n = 35), LMS (n = 55) and UPS (n = 45) profiled by mass spectrometry and (b) in an independent cohort of DDLPS (n = 29), LMS (n = 20) and UPS (n = 45). Significance was determined by one-way ANOVA with Tukey's multiple comparisons test, ns = non significant, *p<0.01, **p<0.001, ***p<0.0001 (c) Representative examples of IHC MMP14 protein staining in DDLPS, LMS and UPS, x20 magnification.
