# Supplementary material for: Clinical Implications and Molecular Features of Extracellular Matrix Networks in Soft Tissue Sarcomas
Source: Clin Cancer Res. 2024 May 29;30(15):3229–42. doi: 10.1158/1078-0432.CCR-23-3960 (PMC11292195; doi:10.1158/1078-0432.CCR-23-3960)
Supplement: Supplementary Figure S3 — Expression of matrisome proteins in a desmoid tumour cohort (n=37) split into 3 groups based on the % tumour content. [file ccr-23-3960_supplementary_figure_s3_suppsf3.pdf]

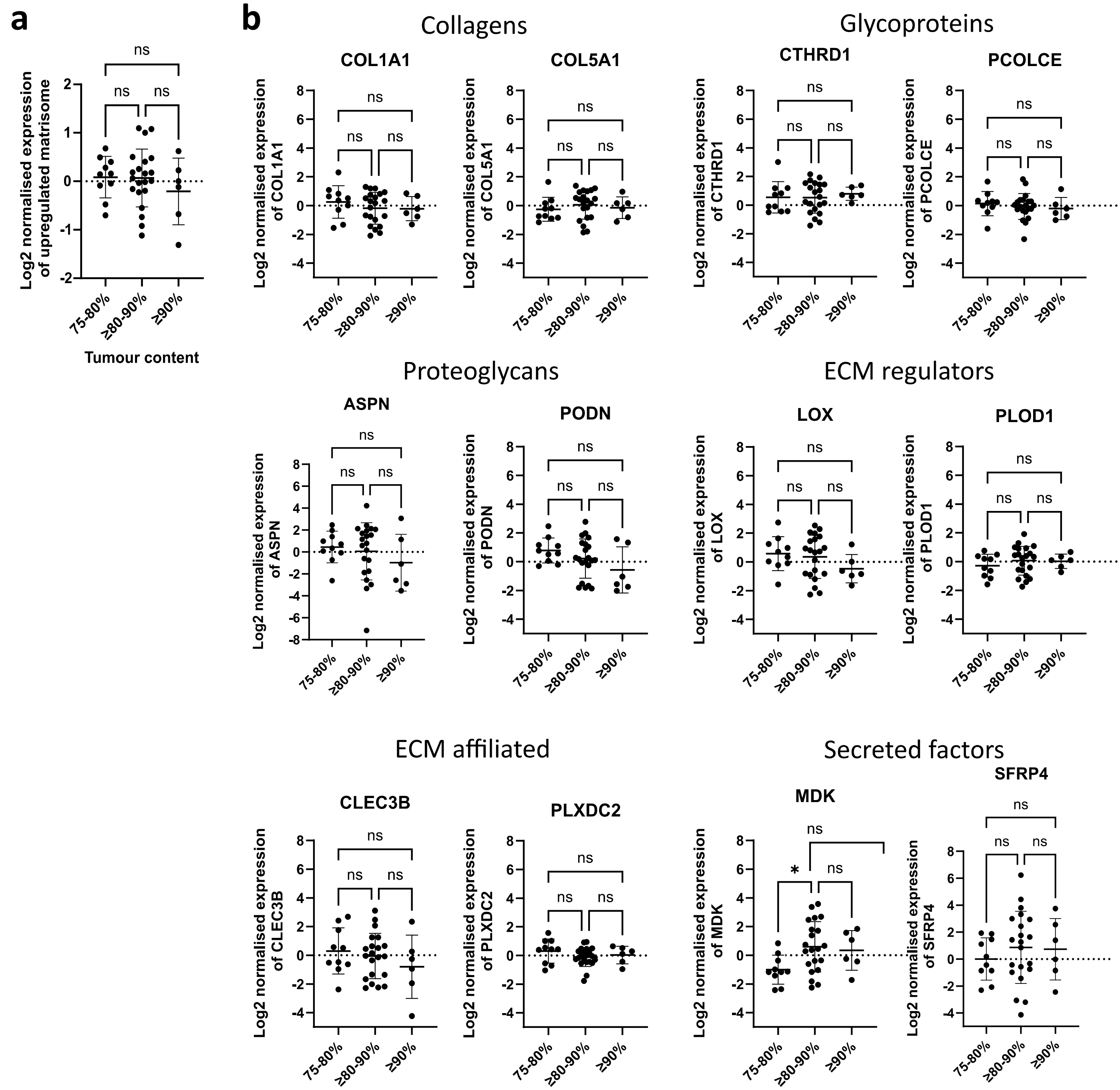

Supplementary Figure S3. Expression of matrisome proteins in a desmoid tumour cohort (n = 37) split into 3 groups based on the % tumour content. (a) Median log2 normalised expression of matrisome proteins enriched in desmoid tumours when classified by percentage tumour content (75-80%, ≥80-90% and ≥90%). (b) Log2 normalised expression of exemplar matrisome proteins from collagen, glycoprotein, proteoglycan, ECM regulators, ECM-affiliated proteins and secreted factors classes, ns = non significant, *p<0.05.
