# Supplementary material for: Clinical Implications and Molecular Features of Extracellular Matrix Networks in Soft Tissue Sarcomas
Source: Clin Cancer Res. 2024 May 29;30(15):3229–42. doi: 10.1158/1078-0432.CCR-23-3960 (PMC11292195; doi:10.1158/1078-0432.CCR-23-3960)
Supplement: Supplementary Figure S4 — Differentially expressed matrisome proteins in synovial sarcoma (SS) cases (n=42) that either received preoperative treatment or were untreated. [file ccr-23-3960_supplementary_figure_s4_suppsf4.pdf]

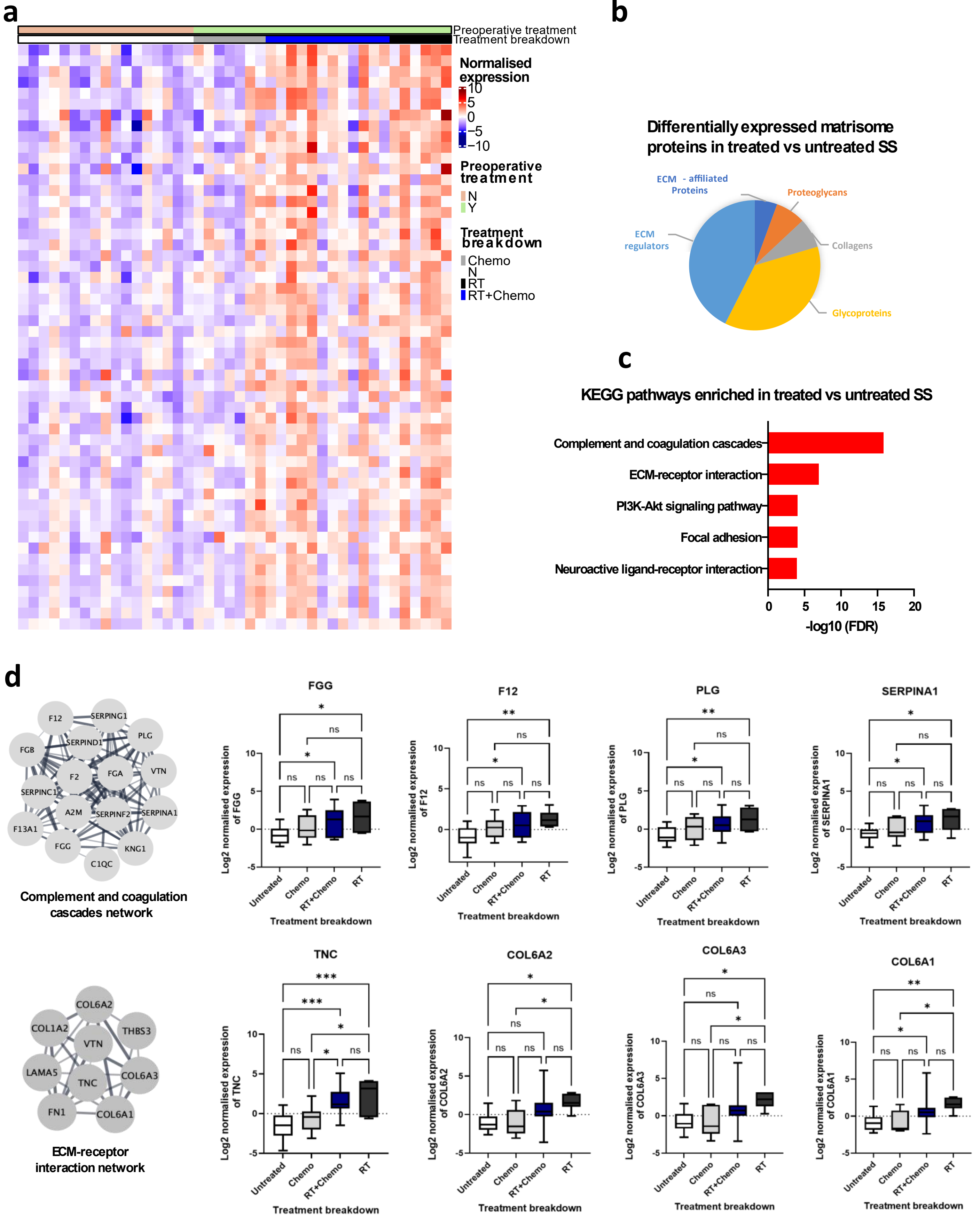

Supplementary Figure S4. Differentially expressed matrisome proteins in synovial sarcoma (SS) cases (n=42) that either received preoperative treatment or were untreated. (a) Heatmap of 54 matrisome proteins significantly upregulated in treated samples compared to untreated samples (fold change > 2, false discovery rate < 0.01). Samples in the heatmap are arranged by treatment type (untreated, chemotherapy alone (Chemo), radiotherapy and chemotherapy (RT+Chemo), and radiotherapy alone (RT)). (b) Breakdown of 54 upregulated matrisome into 6 matrisome classes. (c) Over-representation analysis of upregulated KEGG pathways in SS cases that received preoperative treatment compared to untreated cases. (d) Protein-protein interaction networks constructed using STRINGdb, including KEGG pathway annotations. Box plots show log2 normalised expression of selected matrisome proteins within each of the two KEGG pathway annotations in SS cases split by treatment type. ns = non significant, *p<0.05, **p<0.01, ***p<0.001. SS = synovial sarcoma, ECM = extracellular matrix.
