# Supplementary material for: Clinical Implications and Molecular Features of Extracellular Matrix Networks in Soft Tissue Sarcomas
Source: Clin Cancer Res. 2024 May 29;30(15):3229–42. doi: 10.1158/1078-0432.CCR-23-3960 (PMC11292195; doi:10.1158/1078-0432.CCR-23-3960)
Supplement: Supplementary Figure S5 — Assessment of leiomyosarcoma (LMS) extracellular matrix (ECM) solution and its effects of cell migration. [file ccr-23-3960_supplementary_figure_s5_suppsf5.pdf]

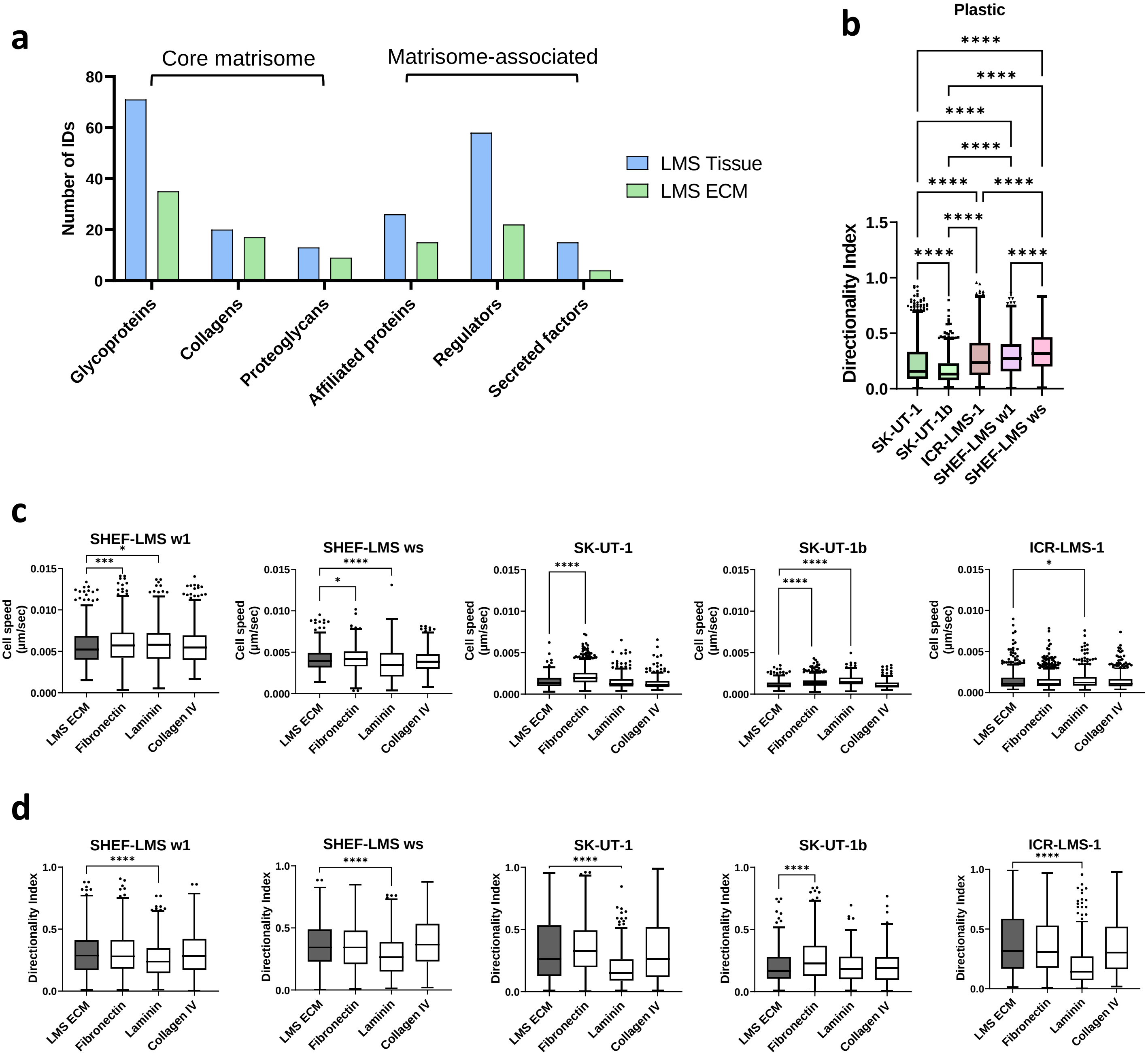

Supplementary Figure S5. Assessment of leiomyosarcoma (LMS) extracellular matrix (ECM) solution and its effects of cell migration. (a) Bar charts showing the breakdown of number of protein IDs in matrisome classes for LMS tumours and LMS ECM solution. (b) Box plots showing the directionality index on plastic in GFP+ SK-UT-1 (n = 910), SK-UT-1b (n = 495), ICR-LMS-1 (n = 528), SHEF-LMS w1 (n = 832) and SHEF-LMS ws (n = 887) cells over 18 h. Significance is shown following Kruskal Wallis tests with Dunn's multiple testing correction, **** $p < 0.0001$. (c) Comparison of cell speed and (d) directionality indices in leiomyosarcoma (LMS) cell lines plated on a coating of LMS ECM and purified fibronectin, laminin or collagen IV. The GFP+ cells were tracked for 18 h. Data were pooled from three independent experiments for all graphs. Boxes indicate the 25th, median, and 75th percentile, with whiskers extending from the 25th percentile-(1.5*IQR) to the 75th percentile+(1.5*IQR), and outliers plotted as points. Significance is shown following Kruskal Wallis tests with Dunn's multiple testing correction, *$p< 0.05$, ***$p< 0.001$ ****$p< 0.0001$.
