# Supplementary material for: Clinical Implications and Molecular Features of Extracellular Matrix Networks in Soft Tissue Sarcomas
Source: Clin Cancer Res. 2024 May 29;30(15):3229–42. doi: 10.1158/1078-0432.CCR-23-3960 (PMC11292195; doi:10.1158/1078-0432.CCR-23-3960)
Supplement: Supplementary Figure S6 — Identification of LCP1 as a candidate prognostic factor in LMS. [file ccr-23-3960_supplementary_figure_s6_suppsf6.pdf]

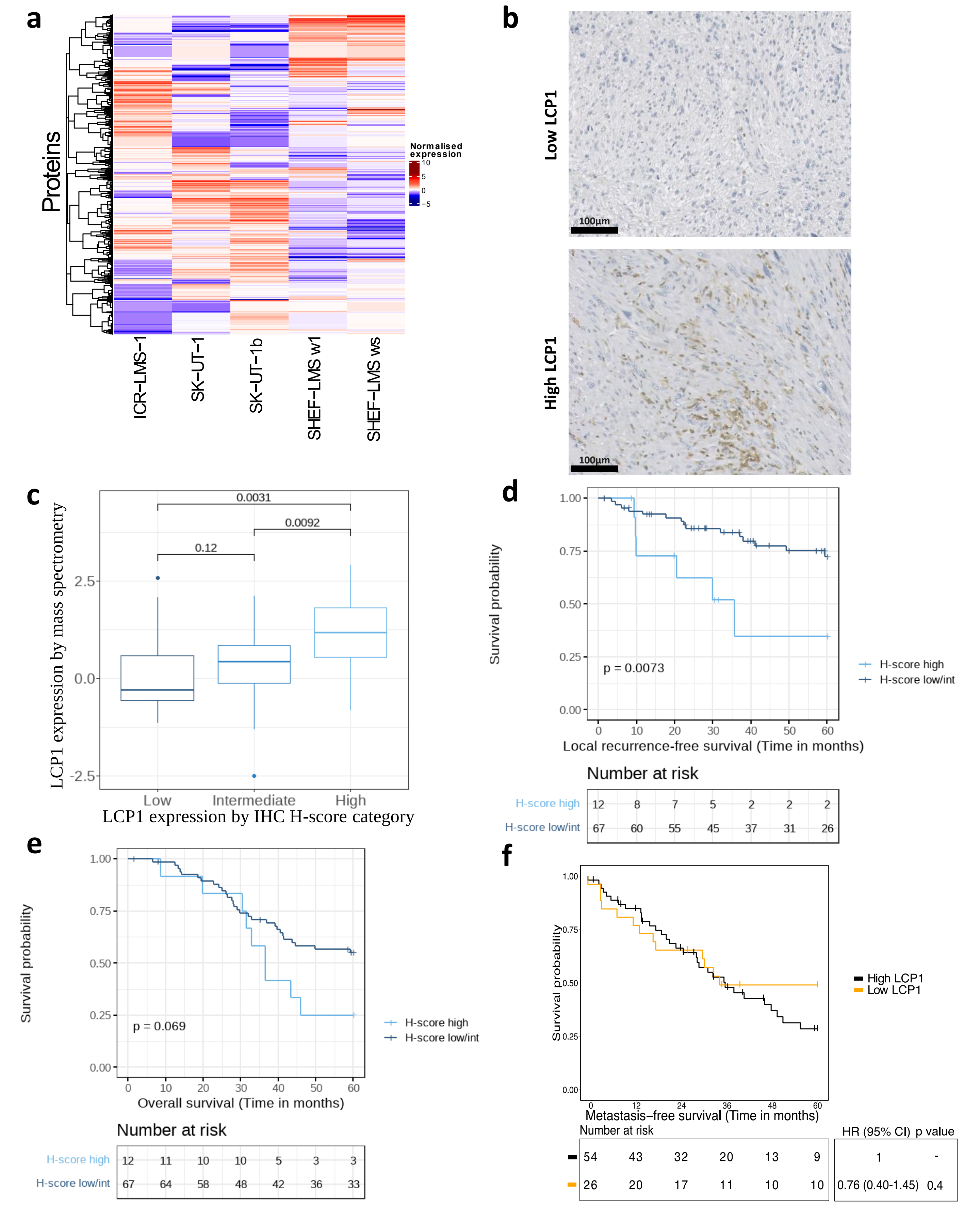

Supplementary Figure S6. Identification of LCP1 as a candidate prognostic factor in LMS. (a) Proteomic characterisation of five LMS cell lines by mass spectrometry analysis of cell lysates (b) representative images of low and high IHC LCP1 staining, x20 magnification (c) correlation of LCP1 protein expression as defined by mass spectrometry and LCP1 IHC H-score categories, significance assessed by Mann Whitney U test Kaplan-Meier plots of (d) local-recurrence free survival and (e) overall survival with stratification by LCP1 IHC H-Score high and LCP1 IHC H-score low+intermediate, p-values were determined by log-rank test (f) Kaplan-Meier plots of metastasis-free survival with stratification by LCP1 proteomics tertiles in 80 LMS patients. Low LCP1 group contains patients with lower tertile (≤ -0.23 LCP1 expression), whilst high LCP1 group is made up of intermediate and high tertiles (> -0.23 LCP1). Hazard ratio (HR), 95% confidence intervals (CI) and p-values were determined by univariate Cox regression with a two-sided Wald test.
