# Supplementary material for: Clinical Implications and Molecular Features of Extracellular Matrix Networks in Soft Tissue Sarcomas
Source: Clin Cancer Res. 2024 May 29;30(15):3229–42. doi: 10.1158/1078-0432.CCR-23-3960 (PMC11292195; doi:10.1158/1078-0432.CCR-23-3960)
Supplement: Supplementary Figure S7 — Clinical characterisation of DDLPS subgroups. [file ccr-23-3960_supplementary_figure_s7_suppsf7.pdf]

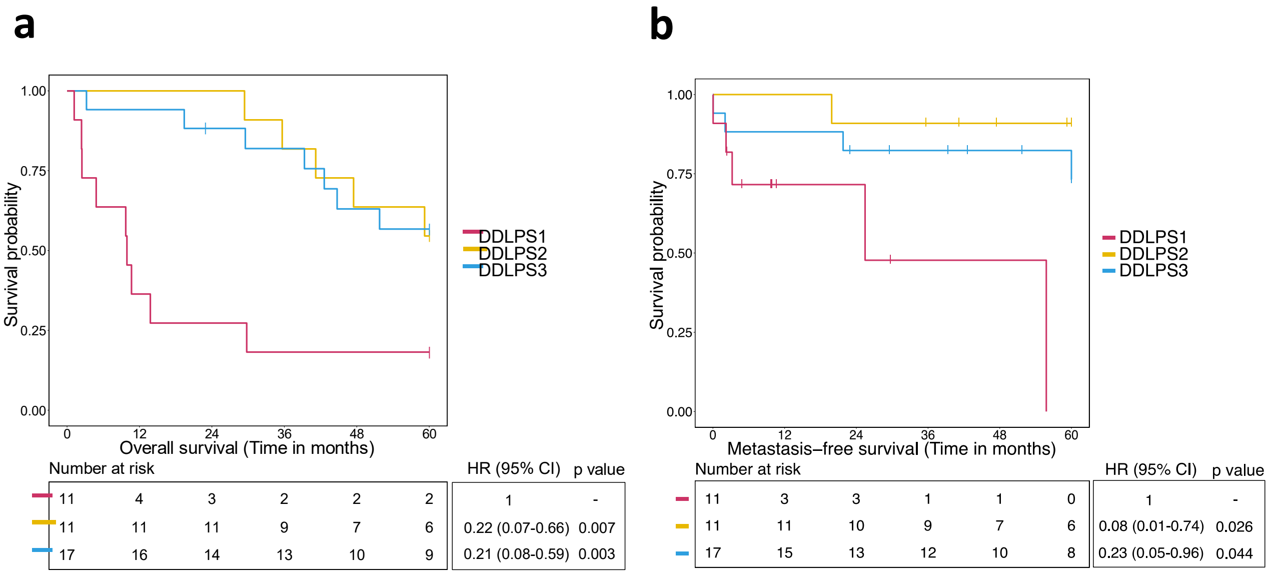

Supplementary Figure S7. Clinical characterisation of DDLPS subgroups. Kaplan-Meier plots of (a) overall survival and (b) metastasis-free survival with stratification by DDLPS subgroups. Hazard ratio (HR), 95% confidence intervals (CI) and p-values were determined by univariate Cox regression with a two-sided Wald test.
