# Supplementary material for: Clinical Implications and Molecular Features of Extracellular Matrix Networks in Soft Tissue Sarcomas
Source: Clin Cancer Res. 2024 May 29;30(15):3229–42. doi: 10.1158/1078-0432.CCR-23-3960 (PMC11292195; doi:10.1158/1078-0432.CCR-23-3960)
Supplement: Supplementary Figure S8 — Association of the proteoglycan gene expression score with survival outcomes in The Cancer Genome Atlas sarcoma (TCGA- SARC) cohort. [file ccr-23-3960_supplementary_figure_s8_suppsf8.pdf]

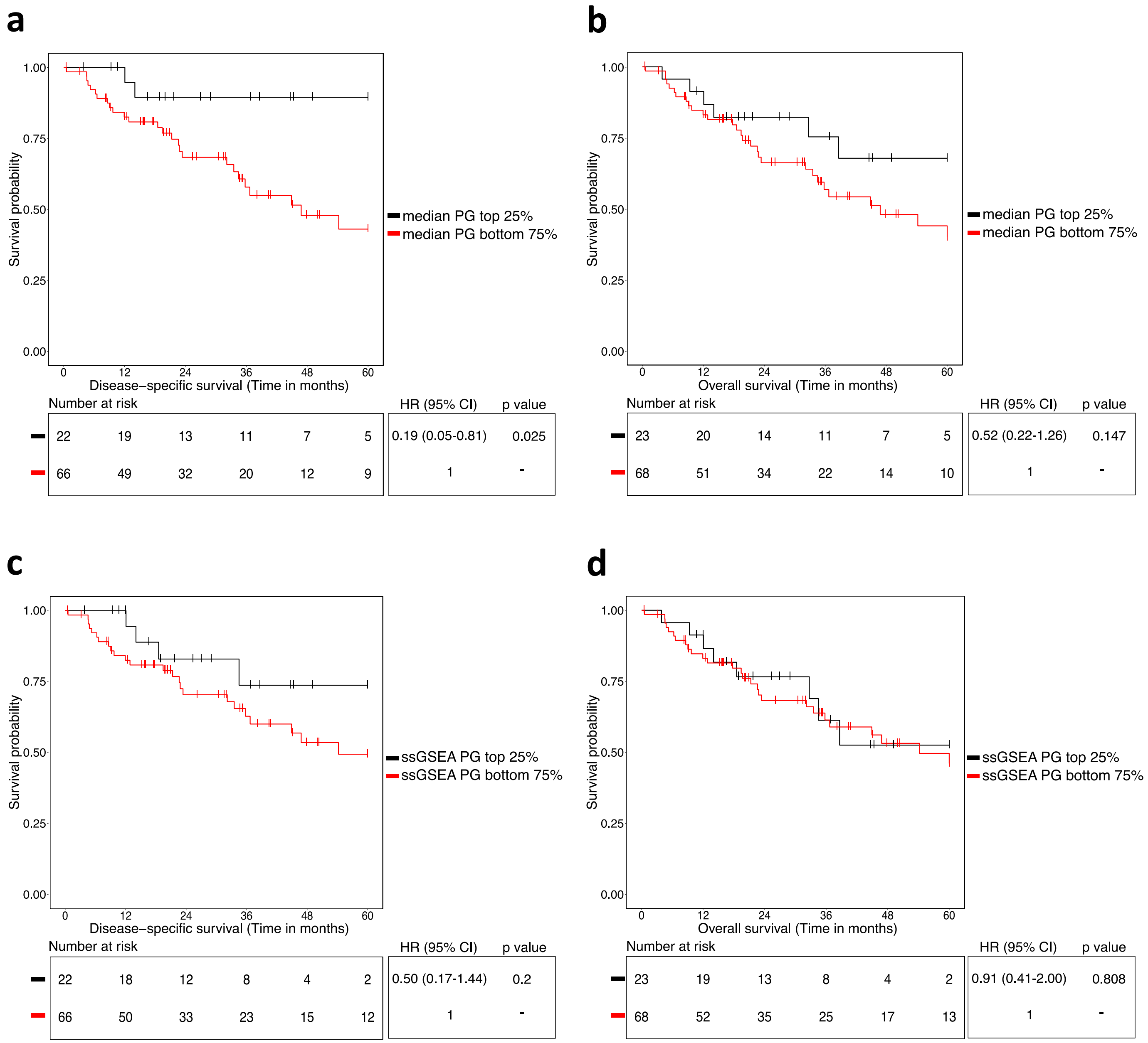

Supplementary Figure S8. Association of the proteoglycan gene expression score with survival outcomes in The Cancer Genome Atlas sarcoma (TCGA- SARC) cohort. Kaplan-Meier plots of (a) disease-specific survival (DSS) and (b) overall survival (OS) with stratification by proteoglycan (median score) quartile expression in a combined n = 91 UPS and DDLPS cohort. Kaplan-Meier plots of (c) disease-specific survival (DSS) and (d) OS with stratification by proteoglycan (ssGSEA score) quartile expression in a combined n = 91 UPS and DDLPS cohort. Hazard ratio (HR), 95% confidence intervals (CI) and p-values were determined by univariate Cox regression with a two-sided Wald test. UPS = undifferentiated pleomorphic sarcoma, DDLPS = dedifferentiated liposarcoma sarcoma, ssGSEA = single-sample Gene Set Enrichment Analysis.
