# Supplementary material for: Clinical Implications and Molecular Features of Extracellular Matrix Networks in Soft Tissue Sarcomas
Source: Clin Cancer Res. 2024 May 29;30(15):3229–42. doi: 10.1158/1078-0432.CCR-23-3960 (PMC11292195; doi:10.1158/1078-0432.CCR-23-3960)
Supplement: Supplementary Methods S1 — Supplemental Methods [file ccr-23-3960_supplementary_methods_s1_suppsm1.docx]

**Supplementary methods**

Processing and analysis of proteomic data from patient specimens

*Processing of raw proteomic data*

All data was processed using custom R scripts v.4.1.1 or later. Proteins identified in <75% of samples were removed. To address the issue of missing data, the remaining missing data was imputed using k-nearest neighbours (k-NN) algorithm in the impute package in R(1). Each sample was divided by corresponding reference sample within the same tandem mass tag set. To remove batch effects, normalisation was performed by log2 transformation, median centring across samples and z-scoring of proteins within each sample. For the sub-cohort and subtype-specific analyses, the raw proteomic data was renormalised to include only samples of interest.

*Clustering*

The processed matrisome and adhesome dataset was visualised using two-way unsupervised clustering based on Pearson’s correlation coefficient and using a dimension reduction by uniform manifold approximation and projection (UMAP)(2). The clustering was visualised with an annotated heatmap plotted using the ComplexHeatmap package(3). The UMAP analysis was performed with the optimal number of 6 neighbours, and the default settings were used for the rest of the parameters. Consensus clustering using the ConsensusClusterPlus package(4) in R was performed to generate stable and robust clustering in DDLPS-specific analyses of matrisome and adhesome. Consensus clustering was conducted using an agglomerative hierarchical clustering algorithm, with Spearman correlation distance and average linking. Protein and sample resampling were set at 80%, and consensus clustering was performed for up to 10 clusters (k). The optimal value of k was determined by inspecting consensus matrices, the cluster tracking plot and the consensus cumulative distribution function (CDF) plot. The statistical significance of the clusters was confirmed using the SigClust package(5) in R with hard thresholding and 1000 sample simulations (p < 0.05).

*Differential expression analysis*

Differentially expressed proteins (DEPs) were identified using significance analysis of microarrays (SAM) with the samr package(6) in R. Normalised and imputed datasets were analysed with two-class unpaired tests based on Student's t-test statistic with 100 permutations for comparisons between two sets of samples. In each comparison, the delta value was selected as the threshold for significance at which the median false discovery rate (FDR) was less than 0.01 and fold change ≥ 2 for upregulated DEPs.

*Protein-protein interaction network analysis*

Protein-protein interaction (PPI) networks were built by querying matrisome and adhesome proteins of interest against the Search Tool for the Retrieval of Interacting Genes/Proteins database (STRINGdb)(7). Only known interactions from curated databases, experimental evidence and literature searches were used as active interaction sources. A default medium confidence cut-off score of 0.4 in the network was applied; the nodes represent proteins with at least one interaction, and the edges represent interactions between the proteins. The resulting networks were visualised and annotated in Cytoscape v3.10.0(8).

*Matrisome and adhesome networks correlation analysis*

To assess which matrisome and adhesome proteins are co-regulated in STS, Pearson’s correlation coefficients were calculated in R for all possible pairwise combinations of matrisome and adhesome proteins. Hierarchical clustering of the resultant similarity matrix was performed and visualised as a heatmap in R. Distinct clusters of co-regulated matrisome and adhesome proteins were identified by the ConsensusClusterPlus(4) and SigClust(5) packages in R.

*Overrepresentation analysis*

Overrepresentation analyses were performed using the online tool g: Profiler (v.e110_eg57_p18_4b54a898)(9) against the Reactome database(10). Benjamini–Hochberg FDR method was applied for multiple testing correction with 0.01 FDR threshold. Proteins present in the dataset were used as the background for overrepresentation analysis.

Proteomic analysis of LMS tumours, cell lines and ECM solution

*Sample preparation of frozen tumours and ECM solution*

For protein digestion, 1 mg of frozen tumour specimen was used. For LMS ECM solution characterisation, 100 μl of the solution was solidified at 37 °C for 1 h prior to the protein digestion. Then, all samples were homogenised in 8 M urea (Sigma-Aldrich) and 100 mM ammonium bicarbonate (ABC, Sigma-Aldrich) in Precellys Evolution at 6800 rpm (cycles of 20 sec, 30 sec break in between cycles). Protein concentration of each sample was measured with a bicinchoninic acid (BCA) assay (Thermo Fisher Scientific). 20 μg of protein was reduced with 10 mM dithiothreitol (Sigma-Aldrich) at 56 °C for 40 min and then alkylated with 25 mM iodoacetamide (Sigma-Aldrich) at 25 °C for 30 min in the dark. Samples were diluted with 100 mM ABC to 2 M urea concentration. The protein sample was digested with trypsin at a ratio 1:25 μg per sample at 37 °C overnight, the samples were desalted using Pierce C18 spin columns (Thermo Fisher Scientific), dried by SpeedVac and dissolved in 2% acetonitrile (ACN, Fisher Scientific), 0.1% formic acid (FA, Honeywell).

*Sample preparation of LMS cell lines*

SK-UT-1, SK-UT-1b, ICR-LMS-1, SHEF-LMS w1 and SHEF-LMS ws cells were lysed with 8 M urea on ice. Protein concentration was estimated with BCA assay. For each sample 40 µg of total protein was reduced with 10 mM of dithiothreitol at 56 °C for 40 minutes and then alkylated with 55 mM iodoacetamide at 25 °C for 30 min in the dark. After diluting to a final concentration of 2 M urea and 100 mM ABC, samples were digested with trypsin at a ratio of 1:100 μg per sample at 37 °C overnight. The acidified digests were desalted using Sep-Pak C18 Plus cartridge (Waters) and dried in a SpeedVac concentrator (Thermo Fisher Scientific).

*Liquid chromatography and mass spectrometry*

For liquid chromatography-tandem mass spectrometry (LC-MS/MS) analysis, samples were dissolved in Buffer A (2% ACN, 0.1% FA), spiked with iRT calibration mix (Biognosys AG) and analysed on an Agilent 1260 HPLC system coupled to a TripleTOF 5600+ mass spectrometer with NanoSource III (AB SCIEX). Sequential window acquisition of all theoretical mass spectra (SWATH) mass spectrometry data was acquired using two μg of peptides for each sample which was loaded onto a ZORBAX C18 (Agilent Technologies) trap column and separated with an integrated manually pulled tip packed with Reprosil Pur C18AQ beads (3 μm, 120 Å particles, Dr. Maisch) with a linear gradient of 2 – 40% of Buffer B (98% ACN, 0.1% FA). Full-profile MS scans were acquired in the mass range of m/z 340–1400 in positive ion mode. Precursor isolation windows had a fixed size of 25 Da across the mass range of m/z 350–1250 with 1 Da overlap. MS/MS scans were acquired in the m/z 100–1500 mass range. All SWATH data were analysed against a publicly available pan-human library using DIA-NN (version 1.8)(11), and trypsin was specified as the cleavage enzyme. The spectral library was refined using the dataset with 0.01 FDR to generate an in-silico library, which was used to reanalyse the data. The subsequent report was filtered at a q-value of 0.01 for both precursor and proteins. Each sample was analysed with two technical replicates, and a protein was considered expressed if it was detected in at least one of the technical replicates. Proteins were annotated as matrisome according to MatrisomeDB(12).

Generation of ICR-LMS-1 cell line

ICR-LMS-1 was established from a tumour of the uterine LMS (uLMS) patient-derived xenograft (PDX) model. The PDX was derived from a baseline biopsy from a grade 3 pelvic LMS tumour. Prospective collection of biopsy and implantation into mice was approved as part of the Royal Marsden Hospital GEMMK trial (NCT03123276) (CCR 4541, REC 17/LO/0767). To dissociate single cells, PDX tumour tissue was minced and digested in dissociation media (DMEM/Ham’s F12 1:1 + 15 mM 4-(2-hydroxyethyl)-1-piperazineethanesulfonic acid (HEPES), 0.5 mg/mL collagenase (Sigma Aldrich), 0.1 mg/mL DNase I (Sigma Aldrich), 10 ng/mL epidermal growth factor (EGF) (Peprotech), 0.1x insulin-transferrin-selenium A (Gibco), 10 µg/mL hydrocortisone (Sigma Aldrich), 10 μM Y-27632, 0.1 mg/mL hyaluronidase (Sigma Aldrich), 5% FBS, and 0.5% penicillin/streptomycin) at 37 ºC for 2 hours on a rotor at 100 rpm. The dissociated tissue was centrifuged at 1400 rpm for 5 minutes and then washed in PBS supplemented with 10 μM Y-27632. Red blood cells (RBCs) were lysed with RBC lysis buffer (Invitrogen) for 1 minute, after which remaining cells were washed with PBS supplemented with 10 μM Y-27632, centrifuged at 1400 rpm for 5 minutes and incubated in 0.05% trypsin-ethylenediaminetetraacetic acid (EDTA) (Gibco) with 10 μM Y-27632 at 37 ºC for 8 minutes. Cells were then incubated in a 1:1 mix of DNase solution (1 mg/mL DNase I and 10 μM Y-27632 in PBS) and Y-media (DMEM:Ham’s F12 1:1 + 15 mM HEPES, 1% L-glutamine, 5 μg/mL insulin (Sigma Aldrich), 0.4 μg/mL hydrocortisone, 10 ng/mL EGF, 250 ng/mL amphotericin (ThermoFisher Scientific), 9.62 ng/mL cholera toxin (Sigma Aldrich), 5 μM Y-27632, 10% FBS, and 0.5% penicillin/streptomycin). Cell suspensions were pelleted by centrifuging at 1400 rpm for 5 minutes then washed in PBS with 10 μM Y-27632 followed by resuspension in Y-media. Cells were then passed through a 70 µm strainer, counted and mouse cell depleted. Mouse cell depletion was achieved by suspending cell pellets in 1x magnetic-activated cell sorting (MACS) buffer, diluted in PBS from a stock solution (20x MACS buffer; 5 g bovine serum albumin (BSA) (Sigma Aldrich), 4 mL 0.5 M EDTA (Sigma Aldrich) in 50 mL PBS), supplemented with 10 μM Y-27632. Mouse cells were labelled in MACS buffer by adding magnetic microbead conjugated antibodies via a Mouse Cell Depletion Kit (Miltenyi Biotec 130-104-694) according to manufacturer’s instructions. The cell solution was incubated on ice for 15 minutes after which the solution was passed through a Quadro MACS magnet LS column (Miltenyi Biotec). The column was washed twice with 1x MACS buffer and the column was then discarded. The resulting flow through contained human enriched uLMS cells, which were pelleted and then resuspended in Y-media for cell counting and subsequent cell culture. Mouse-depleted uLMS cells were passaged 10 times in continuous culture for a period of approximately 16 weeks, at which point these cells were labelled ICR-LMS-1.

H2B-GFP Lentiviral plasmid production, cell transfection and transduction

Lentiviruses were produced by co-transfecting HEK-293T cells with 8 μg PGK-H2B-GFP (Addgene # 21210) and with 4 μg pMD2.G (Addgene #12259) and 4 μg psPAX2 (Addgene #12260) vectors(13). After 96 h, the viral supernatant was harvested and filtered using a 0.45 μm pore filter. SK-UT-1 (150,000 cells/well), SHEF-LMS w1 (100,000 cells/well) and SHEF-LMS ws (100,000 cells/well) were plated in 6 well plates. After 24 h, the medium was aspirated and replaced with twofold serial dilutions of medium containing lentivirus (ranging from 1:2 to 1:8) and a final concentration of 8 μg/ml polybrene (Sigma-Aldrich), to enhance transduction. The transduced cells were enriched for GFP+ cells with fluorescence-activated cell sorting.

Identification of proteoglycan protein prognostic score and its validation in The Cancer Genome Atlas sarcoma (TCGA-SARC) cohort

*Matrisome gene set prognostic scores*

All human matrisome gene sets were from the Molecular Signatures Database (MSigDB) v2023.1.Hs(14). To develop patient-specific median scores, the median protein expression within different matrisome gene sets was calculated for each patient. To stratify patients in high and low groups for survival analyses, the median value across the whole cohort for each separate gene set was used as a cut-off.

*Proteoglycan protein ssGSEA prognostic score*

The human proteoglycan gene set was obtained from the Molecular Signatures Database (MSigDB) v2023.1.Hs(14). To develop patient-specific single sample GSEA (ssGSEA) scores (15), ssGSEA (v10.1.0) was performed on the GenePattern public server. Gene sets database file c2.cgp.v2023.2, rank normalisation and a weighting exponent of 0.75 were used to assess the enrichment of proteoglycan gene set with at least 10 genes. The median value of enrichment scores was used as a cut-off to stratify patients in high and low ssGSEA proteoglycan groups for survival analyses.

*Survival analyses – proteomic data*

The association of biomarker(s) with survival outcome were evaluated based on Kaplan–Meier survival estimates and multivariable Cox regression analyses adjusted for standard clinicopathological variables. Local recurrence-free survival (LRFS) was defined as the time from primary disease surgery to radiologically confirmed local recurrence or death, metastasis-free survival (MFS) defined as the time from primary disease surgery to radiologically confirmed metastatic disease or death and overall survival (OS) defined as the time from primary disease surgery to death from any cause. Clinical data was censored at 5 years, and patients who did not have events were censored at their last follow-up. The significance of differential survival was evaluated by Wald tests.

*The Cancer Genome Atlas sarcoma (TCGA-SARC) cohort*

The RNA sequencing raw counts of 47 DDLPS and 44 UPS, along with the clinicopathological features, were downloaded from the public database Cancer Genome Atlas Program (TCGA) at the Genomic Data Commons Portal (https://portal.gdc.cancer.gov/projects/TCGA-SARC). The dataset was analysed using the package ‘DEseq2’ (ver.1.42.0) in the R environment (4.2.0) to study the RNA expression of differentially expressed genes. The datasets were normalised using the median of ratios by size factors through package ‘DEseq2’ to calculate the accumulated gene score(16). The length of expressed transcripts was downloaded from Ensembl (https://www.ensembl.org) as the reference with package ‘biomaRt’ (ver.2.58.0). The raw counts were divided by the transcriptomic length (in kb) and then normalised with the scale factor, which is equal to the total transcript divided by 106, to compare multiple samples from different subtypes of sarcomas. Three DDLPS transcriptomes were excluded as a quality control measure in the further analysis due to outlier expression of genes of interest.

*Validation of prognostic proteoglycan score in the TCGA-SARC cohort – median expression score*

Human proteoglycan gene set was obtained from the Molecular Signatures Database (MSigDB) v2023.1.Hs(14). To develop patient-specific scores, the median gene expression within the 11-proteoglycan gene set was calculated for each patient. To stratify patients in high (top 25% of the cohort) and low (bottom 75% of the cohort) groups for survival analyses, the quartile values across the whole cohort were used as a cut-off. Clinical data was censored at 5 years, and patients who did not have events were censored at their last follow-up. The association of the median proteoglycan score with OS and disease-specific survival (DSS) were determined by univariate Cox regression with a two-sided Wald test.

*Validation of prognostic proteoglycan score in the TCGA-SARC cohort – ssGSEA score*

Full gene expression list was filtered to 3,361 genes by selecting gene IDs present in the proteomic dataset. To develop gene expression proteoglycan score, the single sample GSEA (ssGSEA)(15) was performed using ssGSEA (v10.1.0) on the GenePattern public server. Gene sets database file c2.cgp.v2023.2, rank normalisation and a weighting exponent of 0.75 were used to assess the enrichment of proteoglycan gene set with at least 10 genes. The quartile values of enrichment scores were used as a cut-off to stratify patients in high (top 25% of the cohort) and low (bottom 75% of the cohort) ssGSEA proteoglycan groups for survival analyses. The association of the ssGSEA proteoglycan score with DSS and OS were determined by univariate Cox regression with a two-sided Wald test.

**References**

1. Hastie T, Tibshirani R, Narasimhan B, Chu G. impute: Imputation for microarray data. R package version 1.64.0. 2020.

2. McInnes L, Healy J, Saul N, Großberger L. UMAP: Uniform Manifold Approximation and Projection. *J Open Source Softw*. 2018;3(29):861 doi 10.21105/joss.00861.

3. Gu Z, Eils R, Schlesner M. Complex heatmaps reveal patterns and correlations in multidimensional genomic data. *Bioinformatics*. 2016;32(18):2847–9 doi 10.1093/bioinformatics/btw313.

4. Wilkerson MD, Hayes DN. ConsensusClusterPlus: a class discovery tool with confidence assessments and item tracking. *Bioinformatics*. 2010;26(12):1572–3 doi 10.1093/bioinformatics/btq170.

5. Liu Y, Hayes DN, Nobel A, Marron JS. Statistical Significance of Clustering for High-Dimension, Low–Sample Size Data. *J Am Stat Assoc*. 2008;103(483):1281–93 doi 10.1198/016214508000000454.

6. Tusher VG, Tibshirani R, Chu G. Significance analysis of microarrays applied to the ionizing radiation response. *Proceedings of the National Academy of Sciences*. 2001;98(9):5116–21 doi 10.1073/pnas.091062498.

7. Szklarczyk D, Kirsch R, Koutrouli M, Nastou K, Mehryary F, Hachilif R, *et al*. The STRING database in 2023: protein–protein association networks and functional enrichment analyses for any sequenced genome of interest. *Nucleic Acids Res*. 2023;51(D1):D638–46 doi 10.1093/nar/gkac1000.

8. Shannon P, Markiel A, Ozier O, Baliga NS, Wang JT, Ramage D, *et al*. Cytoscape: A Software Environment for Integrated Models of Biomolecular Interaction Networks. *Genome Res*. 2003;13(11):2498–504 doi 10.1101/gr.1239303.

9. Kolberg L, Raudvere U, Kuzmin I, Adler P, Vilo J, Peterson H. g:Profiler—interoperable web service for functional enrichment analysis and gene identifier mapping (2023 update). *Nucleic Acids Res*. 2023;51(W1):W207–12 doi 10.1093/nar/gkad347.

10. Fabregat A, Sidiropoulos K, Garapati P, Gillespie M, Hausmann K, Haw R, *et al*. The Reactome pathway Knowledgebase. *Nucleic Acids Res*. 2016;44(D1):D481–7 doi 10.1093/nar/gkv1351.

11. Demichev V, Messner CB, Vernardis SI, Lilley KS, Ralser M. DIA-NN: neural networks and interference correction enable deep proteome coverage in high throughput. *Nat Methods*. 2020;17(1):41–4 doi 10.1038/s41592-019-0638-x.

12. Shao X, Gomez CD, Kapoor N, Considine JM, Grams C, Gao Y (Tom), *et al*. MatrisomeDB 2.0: 2023 updates to the ECM-protein knowledge database. *Nucleic Acids Res*. 2023;51(D1):D1519–30 doi 10.1093/nar/gkac1009.

13. Boettcher M, Tian R, Blau JA, Markegard E, Wagner RT, Wu D, *et al*. Dual gene activation and knockout screen reveals directional dependencies in genetic networks. *Nat Biotechnol*. 2018;36(2):170–8 doi 10.1038/nbt.4062.

14. Liberzon A, Birger C, Thorvaldsdóttir H, Ghandi M, Mesirov JP, Tamayo P. The Molecular Signatures Database Hallmark Gene Set Collection. *Cell Syst*. 2015;1(6):417–25 doi 10.1016/j.cels.2015.12.004.

15. Barbie DA, Tamayo P, Boehm JS, Kim SY, Moody SE, Dunn IF, *et al*. Systematic RNA interference reveals that oncogenic KRAS-driven cancers require TBK1. *Nature*. 2009;462(7269):108–12 doi 10.1038/nature08460.

16. Love MI, Huber W, Anders S. Moderated estimation of fold change and dispersion for RNA-seq data with DESeq2. *Genome Biol*. 2014;15:550 doi 10.1186/s13059-014-0550-8.
