# Supplementary material for: Clinical Implications and Molecular Features of Extracellular Matrix Networks in Soft Tissue Sarcomas
Source: Clin Cancer Res. 2024 May 29;30(15):3229–42. doi: 10.1158/1078-0432.CCR-23-3960 (PMC11292195; doi:10.1158/1078-0432.CCR-23-3960)
Supplement: Supplementary Table S4 — Statistical comparisons between histological subtypes in cluster 1 (C1). [file ccr-23-3960_supplementary_table_s4_suppst4.docx]

| Supplementary Table S4: Statistical comparisons between histological subtypes in cluster 1 (C1). Significant results are in bold. Kruskal-Wallis test was performed and adjusted p values were obtained with Dunn’s multiple correction tests. AS = angiosarcoma; DDLPS = dedifferentiated liposarcoma; DES = desmoid tumour; LMS = leiomyosarcoma; SS = synovial sarcoma; UPS = undifferentiated pleomorphic sarcoma. | | |
| --- | --- | --- |
|  |  |  |
| **Dunn's multiple comparisons test** | **Summary** | **Adjusted P value** |
| **AS vs. SS** | ******** | **<0.0001** |
| **AS vs. UPS** | ******** | **<0.0001** |
| **DDLPS vs. LMS** | ****** | **0.0016** |
| **DDLPS vs. Other** | ***** | **0.0334** |
| **DDLPS vs. SS** | ******** | **<0.0001** |
| **DDLPS vs. UPS** | ****** | **0.0041** |
| **DES vs. SS** | ******** | **<0.0001** |
| **DES vs. UPS** | ******** | **<0.0001** |
| **LMS vs. SS** | ******** | **<0.0001** |
| **LMS vs. UPS** | ******** | **<0.0001** |
| **Other vs. SS** | ******** | **<0.0001** |
| **Other vs. UPS** | ******** | **<0.0001** |
| **SS vs. UPS** | ******** | **<0.0001** |
| AS vs. DDLPS | ns | >0.9999 |
| AS vs. DES | ns | >0.9999 |
| AS vs. LMS | ns | >0.9999 |
| AS vs. Other | ns | >0.9999 |
| DDLPS vs. DES | ns | 0.0654 |
| DES vs. LMS | ns | >0.9999 |
| DES vs. Other | ns | >0.9999 |
| LMS vs. Other | ns | >0.9999 |
