# Supplementary material for: Clinical Implications and Molecular Features of Extracellular Matrix Networks in Soft Tissue Sarcomas
Source: Clin Cancer Res. 2024 May 29;30(15):3229–42. doi: 10.1158/1078-0432.CCR-23-3960 (PMC11292195; doi:10.1158/1078-0432.CCR-23-3960)
Supplement: Supplementary Table S5 — Summary of multivariable (MVA) analyses assessing association of clinicopathological factors and LCP1 tertiles with local recurrence-free survival (LRFS) and overall survival (OS). Clinicopathological factors which were significantly associated with survival in univariable analyses were included in the MVA model. [file ccr-23-3960_supplementary_table_s5_suppst5.docx]

| Supplementary Table S5. Summary of multivariable (MVA) analyses assessing association of clinicopathological factors and LCP1 tertiles with local recurrence-free survival (LRFS) and overall survival (OS). Clinicopathological factors which were significantly associated with survival in univariable analyses were included in the MVA model. Hazard ratio (HR), 95% confidence intervals (CI) and p-values were determined by Cox regression with a two-sided Wald test. | | | | | | | |
| --- | --- | --- | --- | --- | --- | --- | --- |
|  |  |  |  |  |  |  |  |
|  |  | |  | **Multivariable analysis (LRFS)** | | **Multivariable analysis (OS)** | |
| Variable | Groups | | n | HR (95% CI) | p-value | HR (95% CI) | p-value |
| Grade | 2 (reference) | | 47 | - | - | - | - |
|  | 3 | | 33 | 1.06 (0.39-2.85) | 0.911 | 1.63 (0.78-3.42) | 0.192 |
| Anatomical location | Intra-abdominal/Retroperitoneal/Pelvic (reference) | | 38 | - | - | - | - |
|  |  | Extremity/Trunk | 33 | 0.33 (0.10-1.10) | 0.072 | 0.46 (0.19-1.11) | 0.086 |
|  |  | Uterine | 9 | 0.36 (0.04-3.10) | 0.354 | 0.82 (0.27-2.52) | 0.726 |
| Log [tumour size] (mm) | 4-5 (reference) | | 50 | - | - | - | - |
|  |  | <4 | 12 | 0.89 (0.16-4.95) | 0.896 | 0.88 (0.21-3.67) | 0.860 |
|  | >5 | | 17 | 1.73 (0.56-5.28) | 0.339 | 1.59 (0.70-3.63) | 0.272 |
| Tumour depth |  | Deep (reference) | 66 | - | - | - | - |
|  |  | Superficial | 14 | 0.44 (0.04-4.36) | 0.482 | 0.94 (0.26-3.35) | 0.919 |
| Performance status |  | 0 (reference) | 40 | - | - | - | - |
|  | 1 | | 16 | 3.18 (0.97-10.50) | 0.057 | 3.00 (1.15-7.80) | **0.025** |
|  | 2-3 | | 8 | 1.60 (0.18-14.20) | 0.674 | 13.20 (4.19-41.70) | **1.07E-05** |
|  | unknown | | 16 | 1.08 (0.28-4.22) | 0.909 | 0.99 (0.34-2.85) | 0.980 |
| LCP1 tertile | Intermediate+High tertile (reference) | | 54 | - | - | - | - |
|  | Low tertile | | 26 | 0.23 (0.06-0.82) | **0.023** | 0.37 (0.16-0.86) | **0.020** |
