# Supplementary material for: Clinical Implications and Molecular Features of Extracellular Matrix Networks in Soft Tissue Sarcomas
Source: Clin Cancer Res. 2024 May 29;30(15):3229–42. doi: 10.1158/1078-0432.CCR-23-3960 (PMC11292195; doi:10.1158/1078-0432.CCR-23-3960)
Supplement: Supplementary Table S6 — Clinicopathological characteristics of n=39 dedifferentiated liposarcoma (DDLPS). Summary features of the cohort. For continuous variables, the median, minimum (min) and maximum (max) values are indicated. For categorical variables, count and percentage are shown. [file ccr-23-3960_supplementary_table_s6_suppst6.docx]

| Supplementary Table S6: Clinicopathological characteristics of n=39 dedifferentiated liposarcoma (DDLPS). Summary features of the cohort. For continuous variables, the median, minimum (min) and maximum (max) values are indicated. For categorical variables, count and percentage are shown. | | |
| --- | --- | --- |
|  |  |  |
|  |  |  |
| **Total number of patients n (%)** | - | 39 (100) |
| **Age at excision (years)** | Median | 63 |
|  | Min | 35.1 |
|  | Max | 81.3 |
| **Anatomical site [n (%)]** | Extremity | 2 (5.1) |
|  | Intra-abdominal | 3 (7.7) |
|  | Retroperitoneal | 32 (82.1) |
|  | Trunk | 2 (5.1) |
| **Grade [n (%)]** | 2 | 19 (48.7) |
|  | 3 | 20 (51.3) |
| **Tumour depth [n (%)]** | Deep | 38 (97.4) |
|  | Superficial | 1 (2.6) |
| **Tumour size (mm)** | Median | 190 |
|  | Min | 35 |
|  | Max | 1090 |
| **Tumour margins [n (%)]** | R0 | 9 (23.1) |
|  | R1 | 25 (64.1) |
|  | Rx | 5 (12.8) |
| **Pre-op treatment [n (%)]** | Chemo | 1 (2.6) |
|  | None | 38 (97.4) |
| **Performance status [n (%)]** | 0 | 17 (43.6) |
|  | 1 | 12 (30.8) |
|  | 2 | 2 (5.1) |
|  | 3 | 1 (2.6) |
|  | unknown | 7 (17.9) |
| **Sex [n (%)]** | F | 15 (38.5) |
|  | M | 24 (61.5) |
| **Status at excision [n (%)]** | Local | 36 (92.3) |
|  | Metastatic | 2 (5.1) |
|  | Multifocal | 1 (2.6) |
