# Supplementary material for: Clinical Implications and Molecular Features of Extracellular Matrix Networks in Soft Tissue Sarcomas
Source: Clin Cancer Res. 2024 May 29;30(15):3229–42. doi: 10.1158/1078-0432.CCR-23-3960 (PMC11292195; doi:10.1158/1078-0432.CCR-23-3960)
Supplement: Supplementary Table S7 — Summary of statistical tests to assess the association between clinicopathological features and dedifferentiated liposarcoma (DDLPS) subgroups. [file ccr-23-3960_supplementary_table_s7_suppst7.docx]

| Supplementary Table S7: Summary of statistical tests to assess the association between clinicopathological features and dedifferentiated liposarcoma (DDLPS) subgroups. | | | | | | | | |
| --- | --- | --- | --- | --- | --- | --- | --- | --- |
|  |  |  |  |  |  |  |  |  |
| **Characteristic** | **Category** | **DDLPS subgroup** | | | **Test results** | | | |
|  |  | **DDLPS1** | **DDLPS2** | **DDLPS3** | **Test performed** | **χ-squared** | **Degrees of freedom** | **p-value** |
| Age at excision (years) | median | 63.3 | 64.3 | 62.3 | Kruskal Wallis | 0.85 | 2 | 0.654 |
|  | min | 51.4 | 40.7 | 35.1 |  |  |  |  |
|  | max | 81.3 | 80.3 | 80.9 |  |  |  |  |
| **Tumour size (mm)** | median | 170 | 190 | 270 | Kruskal Wallis | 6.54 | 2 | **0.038** |
|  | min | 70 | 35 | 100 |  |  |  |  |
|  | max | 450 | 410 | 1090 |  |  |  |  |
| Anatomical site | Extremity | 0 | 1 | 1 | Chi-square | 5.91 | 6 | 0.434 |
|  | Intra-abdominal | 2 | 1 | 0 |  |  |  |  |
|  | Retroperitoneal | 8 | 8 | 16 |  |  |  |  |
|  | Trunk | 1 | 1 | 0 |  |  |  |  |
| Grade | 2 | 3 | 7 | 9 | Chi-square | 3.13 | 2 | 0.209 |
|  | 3 | 8 | 4 | 8 |  |  |  |  |
| **Performance status** | 0 | 2 | 9 | 6 | Chi-square | 17.51 | 6 | **0.008** |
|  | 1 | 6 | 1 | 5 |  |  |  |  |
|  | 2-3 | 2 | 1 | 0 |  |  |  |  |
|  | unknown | 1 | 0 | 6 |  |  |  |  |
| **Sex** | F | 7 | 1 | 7 | Chi-square | 7.01 | 2 | **0.030** |
|  | M | 4 | 10 | 10 |  |  |  |  |
| Tumour margins | R0 | 2 | 4 | 3 | Chi-square | 4.66 | 4 | 0.324 |
|  | R1 | 6 | 7 | 12 |  |  |  |  |
|  | Rx | 3 | 0 | 2 |  |  |  |  |
