# Supplementary material for: Clinical Implications and Molecular Features of Extracellular Matrix Networks in Soft Tissue Sarcomas
Source: Clin Cancer Res. 2024 May 29;30(15):3229–42. doi: 10.1158/1078-0432.CCR-23-3960 (PMC11292195; doi:10.1158/1078-0432.CCR-23-3960)
Supplement: Supplementary Table S8 — Summary of univariable (UVA) Cox regression analyses assessing the association of clinicopathological factors and dedifferentiated liposarcoma (DDLPS) subgroups with local recurrence-free survival (LRFS), overall survival (OS) and metastasis-free survival (MFS). [file ccr-23-3960_supplementary_table_s8_suppst8.docx]

| Supplementary Table S8: Summary of univariable (UVA) Cox regression analyses assessing the association of clinicopathological factors and dedifferentiated liposarcoma (DDLPS) subgroups with local recurrence-free survival (LRFS), overall survival (OS) and metastasis-free survival (MFS). Hazard ratio (HR), 95% confidence intervals (CI) and p-values were determined by multivariable Cox regression with a two-sided Wald test. Significant p-values are in bold | | | | | | | | | |
| --- | --- | --- | --- | --- | --- | --- | --- | --- | --- |
|  |  |  |  |  |  |  |  |  |  |
|  |  |  | **Univariable analysis (LRFS)** | | **Univariable analysis (OS)** | | **Univariable analysis (MFS)** | |  |
| Variable | Groups | n | HR (95% CI) | p-value | HR (95% CI) | p-value | HR (95% CI) | p-value |  |
|  | Age | - | 1.03 (0.99-1.06) | 0.134 | 1.05 (1.01-1.09) | **0.025** | 1.02 (0.96-1.07) | 0.569 |  |
| Grade | 3 (reference) | 20 | - | - | - | - | - | - |  |
|  | 2 | 19 | 0.70 (0.33-1.49) | 0.352 | 0.38 (0.15-0.95) | **0.038** | 0.34 (0.09-1.34) | 0.125 |  |
| Log [tumour size] (mm) | >5 (reference) | 29 | - | - | - | - | - | - |  |
|  | ≤5 | 10 | 1.24 (0.52-2.95) | 0.625 | 1.59 (0.64-3.96) | 0.318 | 0.78 (0.16-3.68) | 0.749 |  |
| Tumour margin | R1&R2 (reference) | 26 | - | - | - | - | - | - |  |
|  | R0 | 12 | 1.13 (0.50-2.54) | 0.766 | 0.82 (0.32-2.11) | 0.677 | 1.10 (0.27-4.38) | 0.898 |  |
| Sex | M (reference) | 24 | - | - | - | - | - | - |  |
|  | F | 15 | 1.56 (0.73-3.34) | 0.253 | 1.06 (0.44-2.56) | 0.900 | 4.44 (1.15-17.20) | **0.031** |  |
| Performance status | 0 (reference) | 17 | - | - | - | - | - | - |  |
|  | 1 | 12 | 2.19 (0.92-5.20) | 0.076 | 2.71 (1.01-7.31) | **0.049** | 2.62 (0.70-9.89) | 0.155 |  |
|  | 2-3 | 3 | 0.81 (0.10-6.31) | 0.841 | 2.95 (0.61-14.3) | 0.178 | 1.5e-08 (0-Inf) | 0.999 |  |
|  | unknown | 7 | 1.27 (0.44-3.67) | 0.655 | 1.16 (0.3-4.49) | 0.830 | 0.64 (0.07-5.76) | 0.692 |  |
| DDLPS subgroup | DDLPS1 (reference) | 11 | - | - | - | - | - | - |  |
|  | DDLPS2 | 11 | 0.19 (0.06-0.60) | **0.005** | 0.22 (0.07-0.66) | **0.007** | 0.08 (0.01-0.74) | **0.026** |  |
|  | DDLPS3 | 17 | 0.40 (0.16-0.99) | **0.049** | 0.21 (0.08-0.59) | **0.003** | 0.23 (0.05-0.96) | **0.044** |  |
