# Supplementary material for: Clinical Implications and Molecular Features of Extracellular Matrix Networks in Soft Tissue Sarcomas
Source: Clin Cancer Res. 2024 May 29;30(15):3229–42. doi: 10.1158/1078-0432.CCR-23-3960 (PMC11292195; doi:10.1158/1078-0432.CCR-23-3960)
Supplement: Supplementary Table S9 — Summary of multivariable (MVA) Cox regression analyses assessing the association of clinicopathological factors and dedifferentiated liposarcoma (DDLPS) subgroups with local recurrence-free survival (LRFS), overall survival (OS) and metastasis-free survival (MFS). [file ccr-23-3960_supplementary_table_s9_suppst9.docx]

| Supplementary Table S9: Summary of multivariable (MVA) Cox regression analyses assessing the association of clinicopathological factors and dedifferentiated liposarcoma (DDLPS) subgroups with local recurrence-free survival (LRFS), overall survival (OS) and metastasis-free survival (MFS). Hazard ratio (HR), 95% confidence intervals (CI) and p-values were determined by multivariable Cox regression with a two-sided Wald test. Significant p-values are in bold. | | | | | | | | |
| --- | --- | --- | --- | --- | --- | --- | --- | --- |
|  |  |  |  |  |  |  |  |  |
|  |  |  | **Multivariable analysis (LRFS)** | | **Multivariable analysis (OS)** | | **Multivariable analysis (MFS)** | |
| Variable | Groups | n | HR (95% CI) | p-value | HR (95% CI) | p-value | HR (95% CI) | p-value |
|  | Age | - | 1.04 (0.99-1.08) | 0.107 | 1.05 (0.99-1.1) | 0.080 | 1.00 (0.93-1.08) | 0.932 |
| Grade | 3 (reference) | 20 | - | - | - | - | - | - |
|  | 2 | 19 | 1.18 (0.49-2.84) | 0.707 | 0.71 (0.24-2.11) | 0.534 | 0.35 (0.07-1.82) | 0.213 |
| Log [tumour size] (mm) | >5 (reference) | 29 | - | - | - | - | - | - |
|  | ≤5 | 10 | 0.64 (0.22-1.87) | 0.414 | 0.48 (0.15-1.5) | 0.206 | 0.29 (0.05-1.81) | 0.186 |
| Sex | M (reference) | 24 | - | - | - | - | - | - |
|  | F | 15 | 0.73 (0.30-1.82) | 0.504 | 0.56 (0.20-1.6) | 0.279 | 2.57 (0.55-12) | 0.231 |
| Performance status | 0 (reference) | 17 | - | - | - | - | - | - |
|  | 1 | 12 | 0.76 (0.22-2.68) | 0.672 | 1.53 (0.42-5.52) | 0.516 | - | - |
|  | 2-3 | 3 | 1.01 (0.12-8.56) | 0.990 | 2.21 (0.37-13.2) | 0.387 | - | - |
|  | unknown | 7 | 0.80 (0.23-2.75) | 0.725 | 1.34 (0.25-7.21) | 0.735 | - | - |
| DDLPS subgroup | DDLPS1 (reference) | 11 | - | - | - | - | - | - |
|  | DDLPS2 | 11 | 0.11 (0.02-0.56) | **0.008** | 0.15 (0.03-0.69) | **0.015** | 0.14 (0.01-1.86) | 0.135 |
|  | DDLPS3 | 17 | 0.28 (0.08-0.98) | **0.046** | 0.13 (0.03-0.53) | **0.005** | 0.18 (0.03-1.01) | 0.052 |
