# Supplementary material for: Clinical Implications and Molecular Features of Extracellular Matrix Networks in Soft Tissue Sarcomas
Source: Clin Cancer Res. 2024 May 29;30(15):3229–42. doi: 10.1158/1078-0432.CCR-23-3960 (PMC11292195; doi:10.1158/1078-0432.CCR-23-3960)
Supplement: Supplementary Table S10 — Clinicopathological characteristics of n=92 undifferentiated pleomorphic sarcoma (UPS) and dedifferentiated liposarcoma (DDLPS) cases. Summary features of the cohort. For continuous variables, the median, minimum (min) and maximum (max) values are indicated. For categorical variables, count and percentage are shown. [file ccr-23-3960_supplementary_table_s10_suppst10.docx]

| Supplementary Table S10. Clinicopathological characteristics of n=92 undifferentiated pleomorphic sarcoma (UPS) and dedifferentiated liposarcoma (DDLPS) cases. Summary features of the cohort. For continuous variables, the median, minimum (min) and maximum (max) values are indicated. For categorical variables, count and percentage are shown. | | |
| --- | --- | --- |
|  |  | Number (%) |
|  | n | 92 |
| **Histological subtype** | DDLPS | 39 |
|  | UPS | 53 |
| **Age at excision (years)** | median | 68.6 |
|  | min | 28.2 |
|  | max | 90 |
| **Tumour size (mm)** | median | 120 |
|  | min | 15 |
|  | max | 1090 |
| **Sex** | F | 43 (46.7) |
|  | M | 49 (53.3) |
| **Grade** | 2 | 22 (23.9 |
|  | 3 | 69 (75) |
|  | unknown | 1 (1.1) |
| **Anatomical site** | Extremity | 40 (43.5) |
|  | Head/neck | 4 (4.3) |
|  | Intra-abdominal | 4 (4.3) |
|  | Retroperitoneal | 32 (34.8) |
|  | Trunk | 10 (10.9) |
|  | Pelvic | 2 (2.2) |
| **Tumour depth** | Deep | 81 (88) |
|  | Superficial | 11 (12) |
| **Status at excision** | Local | 89 (96.7 |
|  | Metastatic | 2 (2.2) |
|  | Multifocal | 1 (1.1) |
| **Radiation associated** | No | 87 (94.6) |
|  | Yes | 5 (5.4) |
| **Tumour margins** | R0 | 35 (38) |
|  | R1 | 52 (56.5) |
|  | unknown | 5 (5.5) |
| **Performance status** | 0 | 39 (42.4) |
|  | 1 | 27 (29.3) |
|  | 2 | 6 (6.5) |
|  | 3 | 4 (4.3) |
|  | unknown | 16 (17.4) |
