# Supplementary material for: Clinical Implications and Molecular Features of Extracellular Matrix Networks in Soft Tissue Sarcomas
Source: Clin Cancer Res. 2024 May 29;30(15):3229–42. doi: 10.1158/1078-0432.CCR-23-3960 (PMC11292195; doi:10.1158/1078-0432.CCR-23-3960)
Supplement: Supplementary Table S11 — Statistical association of clinicopathological features with proteoglycan groups. [file ccr-23-3960_supplementary_table_s11_suppst11.docx]

| Supplementary Table S11. Statistical association of clinicopathological features with proteoglycan groups. Abbreviations: F = female; M = male; UPS = undifferentiated pleomorphic sarcoma; DDLPS = dedifferentiated liposarcoma. | | | | | | | |
| --- | --- | --- | --- | --- | --- | --- | --- |
|  |  |  |  |  |  |  |  |
| Variable | | Proteoglycan group | | Test results | | | |
|  |  | Low | High | Test performed | χ-squared | Degrees of freedom | p-value |
| Age at excision (years) | median | 70.0 | 65.1 | Kruskal Wallis | 1.36 | 1 | 0.243 |
|  | min | 28.2 | 35.1 |  |  |  |  |
|  | max | 86.3 | 90.0 |  |  |  |  |
| Tumour size (mm) | median | 110 | 142.5 | Kruskal Wallis | 3.14 | 1 | 0.076 |
|  | min | 15 | 17 |  |  |  |  |
|  | max | 430 | 1090 |  |  |  |  |
| Anatomical site | Extremity | 23 | 17 | chi-square | 8 | 5 | 0.156 |
|  | Head/neck | 2 | 2 |  |  |  |  |
|  | Intra-abdominal | 3 | 1 |  |  |  |  |
|  | Pelvic | 1 | 1 |  |  |  |  |
|  | Retroperitoneal | 10 | 22 |  |  |  |  |
|  | Trunk | 7 | 3 |  |  |  |  |
| **Grade** | 2 | 2 | 20 | chi-square | 19.95 | 1 | **<0.0001** |
|  | 3 | 44 | 25 |  |  |  |  |
| **Performance status** | 0 | 13 | 26 | chi-square | 9.97 | 3 | **0.019** |
|  | 1 | 14 | 13 |  |  |  |  |
|  | 2-3 | 7 | 3 |  |  |  |  |
|  | unknown | 12 | 4 |  |  |  |  |
| **Sex** | F | 31 | 12 | chi-square | 15.76 | 1 | **<0.0001** |
|  | M | 15 | 34 |  |  |  |  |
| **Subtype** | DDLPS | 13 | 26 | chi-square | 7.52 | 1 | **0.006** |
|  | UPS | 33 | 20 |  |  |  |  |
| Tumour margins | R0 | 18 | 17 | chi-square | 0.23 | 1 | 0.892 |
|  | R1 | 26 | 26 |  |  |  |  |
|  | Rx | 2 | 3 |  |  |  |  |
| Tumour depth | Deep | 40 | 41 | chi-square | 0.1 | 1 | 0.748 |
|  | Superficial | 6 | 5 |  |  |  |  |
