# Supplementary material for: Clinical Implications and Molecular Features of Extracellular Matrix Networks in Soft Tissue Sarcomas
Source: Clin Cancer Res. 2024 May 29;30(15):3229–42. doi: 10.1158/1078-0432.CCR-23-3960 (PMC11292195; doi:10.1158/1078-0432.CCR-23-3960)
Supplement: Supplementary Table S12 — Summary of univariable (UVA) and multivariable (MVA) Cox regression analyses assessing the association of clinicopathological factors and median proteoglycan score with overall survival (OS). [file ccr-23-3960_supplementary_table_s12_suppst12.docx]

| Supplementary Table S12. Summary of univariable (UVA) and multivariable (MVA) Cox regression analyses assessing the association of clinicopathological factors and median proteoglycan score with overall survival (OS). Hazard ratio (HR), 95% confidence intervals (CI) and p-values were determined by Cox regression with a two-sided Wald test. I-A = intra-abdominal; RP = retroperitoneal, DDLPS = dedifferentiated liposarcoma and UPS = undifferentiated pleomorphic sarcoma. | | | | | | |
| --- | --- | --- | --- | --- | --- | --- |
|  |  |  |  |  |  |  |
|  |  |  | **Univariable analysis (OS)** | | **Multivariable analysis (OS)** | |
| Variable | Groups | n | HR (95% CI) | p-value | HR (95% CI) | p-value |
| Age |  | 92 | 1.04 (1.02-1.07) | **0.001** | 1.04 (1.00-1.07) | **0.033** |
| Grade | 3 (reference) | 69 | - | - | - | - |
|  | 2 | 22 | 0.44 (0.20-0.93) | **0.031** | 0.67 (0.27-1.69) | 0.395 |
| STS subtype | UPS (reference) | 53 | - | - | - | - |
|  | DDLPS | 39 | 0.81 (0.47-1.42) | 0.467 | 1.08 (0.23-5.02) | 0.925 |
| Anatomical location | Other (reference) | 56 | - | - | - | - |
|  | I-A/RP | 36 | 2.56 (0.80-8.26) | 0.115 | 0.713 (0.14-3.64) | 0.685 |
| Log [tumour size] (mm) | 4-5 (reference) | 39 | - | - | - | - |
|  | >5 | 37 | 1.02 (0.57-1.84) | 0.938 | 1.92 (0.84-4.39) | 0.123 |
|  | <4 | 16 | 0.55 (0.24-1.29) | 0.171 | 0.41 (0.15-1.16) | 0.092 |
| Tumour depth | Deep (reference) | 81 | - | - | - | - |
|  | Superficial | 11 | 0.67 (0.27-1.69) | 0.400 | 0.47 (0.14-1.61) | 0.227 |
| Tumour margin | R1&R2 | 53 | - | - | - | - |
|  | R0 | 38 | 0.82 (0.46-1.43) | 0.476 | 0.84 (0.44-1.61) | 0.598 |
| Sex | M (reference) | 49 | - | - | - | - |
|  | F | 43 | 0.94 (0.54-1.62) | 0.812 | 0.88 (0.45-1.73) | 0.716 |
| Performance status | 0 (reference) | 39 | - | - | - | - |
|  | 1 | 27 | 2.66 (1.35-5.24) | **0.005** | 3.07 (1.41-6.66) | **0.005** |
|  | 2-3 | 10 | 4.21 (1.76-10.1) | **0.001** | 1.96 (0.67-5.75) | 0.221 |
|  | unknown | 16 | 2.70 (1.21-6.02) | **0.015** | 1.61 (0.61-4.27) | 0.341 |
| Median proteoglycan score | Proteoglycan low (reference) | 46 | - | - | - | - |
|  | Proteoglycan high | 46 | 0.50 (0.29-0.88) | **0.015** | 0.39 (0.18-0.85) | **0.017** |
