# Supplementary material for: Clinical Implications and Molecular Features of Extracellular Matrix Networks in Soft Tissue Sarcomas
Source: Clin Cancer Res. 2024 May 29;30(15):3229–42. doi: 10.1158/1078-0432.CCR-23-3960 (PMC11292195; doi:10.1158/1078-0432.CCR-23-3960)
Supplement: Supplementary Table S13 — Summary of univariable (UVA) and multivariable (MVA) Cox regression analyses assessing the association of clinicopathological factors and ssGSEA-derived proteoglycan score with overall survival (OS). [file ccr-23-3960_supplementary_table_s13_suppst13.docx]

| Supplementary Table S13. Summary of univariable (UVA) and multivariable (MVA) Cox regression analyses assessing the association of clinicopathological factors and ssGSEA-derived proteoglycan score with overall survival (OS). Hazard ratio (HR), 95% confidence intervals (CI) and p-values were determined by Cox regression with a two-sided Wald test. I-A = intra-abdominal; RP = retroperitoneal, DDLPS = dedifferentiated liposarcoma and UPS = undifferentiated pleomorphic sarcoma, ssGSEA = single-sample Gene Set Enrichment Analysis. | | | | | | |
| --- | --- | --- | --- | --- | --- | --- |
|  |  |  |  |  |  |  |
|  |  |  | **Univariable analysis (OS)** | | **Multivariable analysis (OS)** | |
| Variable | Groups | n | HR (95% CI) | p-value | HR (95% CI) | p-value |
| Age |  | 92 | 1.04 (1.02-1.07) | **0.001** | 1.04 (1.00-1.07) | **0.032** |
| Grade | 3 (reference) | 69 | - | - | - | - |
|  | 2 | 22 | 0.44 (0.20-0.93) | **0.031** | 0.61 (0.25-1.52) | 0.289 |
| STS subtype | UPS (reference) | 53 | - | - | - | - |
|  | DDLPS | 39 | 0.81 (0.47-1.42) | 0.467 | 1.02 (0.22-4.83) | 0.979 |
| Anatomical location | Other (reference) | 56 | - | - | - | - |
|  | I-A/RP | 36 | 2.56 (0.80-8.26) | 0.115 | 0.73 (0.14-3.8) | 0.711 |
| Log [tumour size] (mm) | 4-5 (reference) | 39 | - | - | - | - |
|  | >5 | 37 | 1.02 (0.57-1.84) | 0.938 | 1.92 (0.84-4.4) | 0.125 |
|  | <4 | 16 | 0.55 (0.24-1.29) | 0.171 | 0.41 (0.15-1.16) | 0.092 |
| Tumour depth | Deep (reference) | 81 | - | - | - | - |
|  | Superficial | 11 | 0.67 (0.27-1.69) | 0.400 | 0.48 (0.14-1.63) | 0.236 |
| Tumour margin | R1&R2 | 53 | - | - | - | - |
|  | R0 | 38 | 0.82 (0.46-1.43) | 0.476 | 0.85 (0.44-1.65) | 0.638 |
| Sex | M (reference) | 49 | - | - | - | - |
|  | F | 43 | 0.94 (0.54-1.62) | 0.812 | 0.93 (0.48-1.83) | 0.838 |
| Performance status | 0 (reference) | 39 | - | - | - | - |
|  | 1 | 27 | 2.66 (1.35-5.24) | **0.005** | 3.03 (1.4-6.56) | **0.005** |
|  | 2-3 | 10 | 4.21 (1.76-10.1) | **0.001** | 2.00 (0.68-5.89) | 0.209 |
|  | unknown | 16 | 2.70 (1.21-6.02) | **0.015** | 1.74 (0.65-4.61) | 0.269 |
| ssGSEA Proteoglycan score | Proteoglycan low (reference) | 46 | - | - | - | - |
|  | Proteoglycan high | 46 | 0.58 (0.33-0.99) | **0.049** | 0.47 (0.22-0.98) | **0.044** |
